# Molecular cloning, heterologous expression, and enzymatic characterization of lysoplasmalogen‐specific phospholipase D from *Thermocrispum* sp.

**DOI:** 10.1002/2211-5463.12131

**Published:** 2016-10-17

**Authors:** Yusaku Matsumoto, Nana Kashiwabara, Takayuki Oyama, Kazutaka Murayama, Hideyuki Matsumoto, Shin‐ich Sakasegawa, Daisuke Sugimori

**Affiliations:** ^1^ Department of Symbiotic Systems Science and TechnologyGraduate School of Symbiotic Systems Science and TechnologyFukushima UniversityJapan; ^2^ Division of Biomedical Measurements and DiagnosticsGraduate School of Biomedical EngineeringTohoku UniversitySendaiJapan; ^3^ Asahi Kasei Pharma Corp.IzunokuniShizuokaJapan

**Keywords:** biochemical characterization, cloning and heterologous expression, head group recognition, lysoplasmalogen‐specific phospholipase D, *Thermocrispum* sp.

## Abstract

Lysoplasmalogen (LyPls)‐specific phospholipase D (LyPls‐PLD) is an enzyme that catalyses the hydrolytic cleavage of the phosphoester bond of LyPls, releasing ethanolamine or choline, and 1‐(1‐alkenyl)‐*sn*‐glycero‐3‐phosphate (lysoplasmenic acid). Little is known about LyPls‐PLD and metabolic pathways of plasmalogen (Pls). Reportedly, Pls levels in human serum/plasma correlate with several diseases such as Alzheimer's disease and arteriosclerosis as well as a variety of biological processes including apoptosis and cell signaling. We identified a LyPls‐PLD from *Thermocrispum* sp. strain RD004668, and the enzyme was purified, characterized, cloned, and expressed using pET24a(+)/*Escherichia coli* with a His tag. The enzyme's preferred substrate was choline LyPls (LyPlsCho), with only modest activity toward ethanolamine LyPls. Under optimum conditions (pH 8.0 and 50 °C), steady‐state kinetic analysis for LyPlsCho yielded *K*_m_ and *k*_cat_ values of 13.2 μm and 70.6 s^−1^, respectively. The ORF of LyPls‐PLD gene consisted of 1005 bp coding a 334‐amino‐acid (aa) protein. The deduced aa sequence of LyPls‐PLD showed high similarity to those of glycerophosphodiester phosphodiesterases (GDPDs); however, the substrate specificity differed completely from those of GDPDs and general phospholipase Ds (PLDs). Structural homology modeling showed that two putative catalytic residues (His46, His88) of LyPls‐PLD were highly conserved to GDPDs. Mutational and kinetic analyses suggested that Ala55, Asn56, and Phe211 in the active site of LyPls‐PLD may participate in the substrate recognition. These findings will help to elucidate differences among LyPls‐PLD, PLD, and GDPD with regard to function, substrate recognition mechanism, and biochemical roles.

**Data Accessibility:**

*Thermocrispum* sp. strain RD004668 and its 16S rDNA sequence were deposited in the NITE Patent Microorganisms Depositary (NPMD; Chiba, Japan) as NITE BP‐01628 and in the DDBJ database under the accession number AB873024. The nucleotide sequences of the 16S rDNA of strain RD004668 and the LyPls‐PLD gene were deposited in the DDBJ database under the accession numbers AB873024 and AB874601, respectively.

**Enzyme:**

EC number EC 3.1.4.4

**Abbreviations**
2ME: 2‐mercaptoethanol
4‐AA: 4‐aminoantipyrine
Cho: choline
COD: choline oxidase
DAGPL(s): diacylglycerophospholipid(s)
DTT: dithiothreitol
Etn: ethanolamine
GDPD: glycerophosphodiester phosphodiesterases
GPC‐CP: glycerophosphocholine cholinephosphodiesterase
GPC: glycerol‐3‐phosphocholine
GPE‐EP: glycerophosphoethanolamine ethanolaminephosphodiesterase
GPE: glycerol‐3‐phospho ethanolamine
IAA: iodoacetamide
LPA: 1‐stearoyl‐2‐hydroxy‐*sn*‐glycero‐3‐phosphate
LPC: 1‐palmitoyl‐2‐hydroxy‐*sn*‐glycero‐3‐phosphocholine
LyPlsA: 1‐(1‐alkenyl)‐*sn*‐glycero‐3‐phosphate
LyPlsCho: 1‐*O‐*1′‐(Z)‐octadecenyl‐2‐hydroxy‐*sn*‐glycero‐3‐phosphocholine (choline lysoplasmalogen)
LyPlsEtn: 1‐*O‐*1′‐(Z)‐octadecenyl‐2‐hydroxy‐*sn*‐glycero‐3‐phosphoethanolamine (ethanolamine lysoplasmalogen)
LyPls: lysoplasmalogen(s)
LyPls‐PLD: lysoplasmalogen‐specific phospholipase D
NGS: next‐generation sequencing
PI: phosphoinositide
PLA_1_: phospholipase A_1_
PLC: phospholipase C
PLD: phospholipase D
PlsA: 2‐acyl‐1‐(1‐alkenyl)‐*sn*‐glycero‐3‐phosphate
Pls: plasmalogen(s)
PMSF: phenylmethylsulfonyl fluoride
POD: peroxidase
POPC: 1‐palmitoyl‐2‐oleoyl‐*sn*‐glycero‐3‐phosphocholine
POPE: 1‐palmitoyl‐2‐oleoyl‐*sn*‐glycero‐3‐phosphoethanolamine
SM: sphingomyelin
TODB: *N,N*‐Bis(4‐sulfobutyl)‐3‐methylaniline, disodium salt

Plasmalogens (Pls) are glycerophospholipids with a vinyl ether bond at the *sn*‐1 position and an ester bond at the *sn*‐2 position. Pls are broadly found in organisms from anaerobic bacteria to invertebrate and vertebrate animals 1. Pls as well as common diacylglycerophospholipids (DAGPLs) are important components of cell membrane. In mammals, Pls constitute 4–32% of the total phospholipid mass, and are particularly enriched in neural tissue, heart, lung, and circulating immune cells 2, 3. Although the functions of Pls have not been fully elucidated, choline Pls (PlsCho) and ethanolamine Pls (PlsEtn) levels in human serum and plasma correlate with a variety of biological processes including apoptosis, cell signaling 4, and various diseases 5, 6, 7. Recently, PlsEtn and PlsCho have been implicated in several diseases such as Alzheimer's disease and arteriosclerosis 5, respectively. More recently, Maeba *et al*. reported that PlsChos, particularly those containing oleic acid (18 : 1) in the *sn*‐2 position, were strongly associated with a wide range of risk factors for metabolic syndrome and atherosclerosis and may therefore be useful biomarkers 7. As interest grows in the development of diagnostic reagent kits for early stage diseases, the Pls‐specific enzymes are receiving increasing attention.

There are several reports on enzymes involved in Pls metabolism. In mammals, LyPls are formed from Pls by the action of phospholipase A_2_ (PLA_2_), which cleaves the *sn*‐2 acyl bond of DAGPLs to release a free fatty acid (FFA) 3. Recently, we also found that phospholipase A_1_ (PLA_1_), which cleaves the *sn*‐1 acyl bond of DAGPLs to release FFA, of *Streptomyces albidoflavus* NA297 (SaPLA_1_) can hydrolyze PlsCho and PlsEtn to yield choline lysoplasmalogen (LyPlsCho) and ethanolamine lysoplasmalogen (LyPlsEtn), respectively 8. In mammals, it is important that removal of the acyl moiety from *sn*‐2 of Pls by the action of PLA_2_ is the only known pathway for generation of lysoplasmalogens (LyPls). In contrast with Pls, LyPls are amphiphilic and are normally maintained at very low levels in cell membranes 3. Wu *et al*. reported lysoplasmalogenase (LyPlsase) identified as an integral membrane protein TMEM86B (EC 3.3.2.2 and EC 3.3.2.5) that catalyses the hydrolytic cleavage of the vinyl ether bond at the *sn*‐1 position of LyPls, forming fatty aldehyde and *sn*‐glycero‐3‐phosphoethanolamine (GPE) or *sn*‐glycero‐3‐phosphocholine (GPC) 1, 9. They also purified and characterized LyPlsase from *Legionella pneumophila* and cloned its gene 3. Moreover, they described that LyPlsase (TMEM86B) is member of the larger YhhN family of proteins which are present in 138 species of eukaryotes and 1205 of bacteria. LyPls can be converted back into Pls by a transacylase 10. LyPls can be further broken down by phospholipase C (PLC) and phospholipase D (PLD) 11, 12, 13. Wolf and Gross reported that PLC in the cytosolic fraction of canine myocardium can hydrolyze Pls, releasing the head groups, phosphoethanolamine or phosphocholine 14. Wykle and Schremmer reported that lysophospholipase D in brain microsomes can hydrolyze LyPls or 1‐*O‐*1′‐alkyl‐2‐hydroxylglycerophospholipid, releasing ethanolamine (Etn) or choline (Cho) 11; however, this enzyme has not been purified and its gene has not been identified. We recently found that PLD (PLD_684_) from *Streptomyces* sp. NA684 can hydrolyze PlsCho in the presence of 0.1–0.2% (w/v) Triton X‐100 15. To the best of our knowledge, however, no other information is available concerning LyPls‐ or Pls‐specific PLD. Also, little is known about Pls metabolic pathways. Understandably, their enzymes also remain to be characterized.

In the present study, we describe a novel enzyme, LyPls‐specific PLD (LyPLs‐PLD), capable of hydrolyzing LyPls to release the corresponding Cho or Etn (Fig. 1). Here, we report the purification, characterization, molecular cloning of LyPLs‐PLD from *Thermocrispum* sp. strain RD004668, as well as its efficient heterologous production using *Escherichia coli*. The identification of LyPls‐PLD reveals a new pathway for Pls metabolism, in particular, in microorganisms. The characterization of LyPls‐PLD demonstrates differences between PLD, the phosphodiesterase family, and glycerophosphodiester phosphodiesterase (GDPD) [EC 3.1.4.46] with regard to function, substrate recognition mechanism, and biochemical roles.

**Figure 1:**
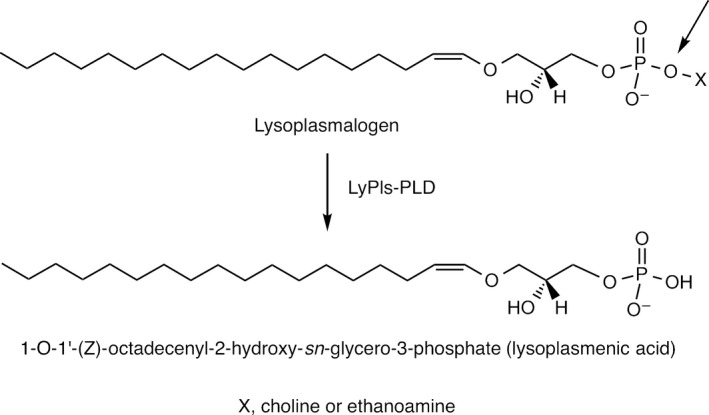
Hydrolysis of LyPls by LyPls‐PLD.

## Results

### Purification and characterization of the wild‐type enzyme

**Figure 2:**
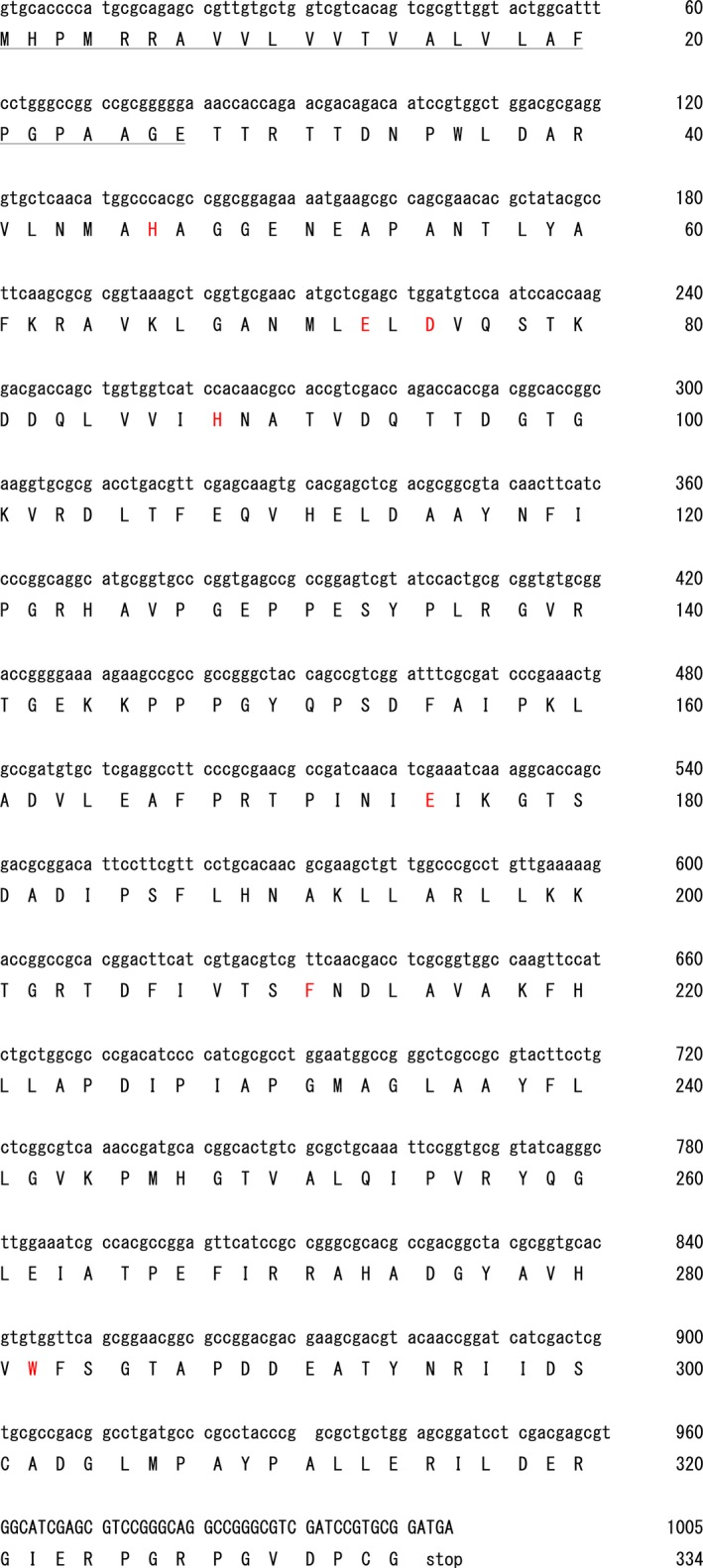
Deduced amino acid sequence of LyPls‐PLD. Red letters, conserved active center amino acids; underline, signal peptide.

The purified wild‐type (WT) enzyme (30.7 μg protein) with specific activity of 50.6 U·mg^−1^ protein was obtained from the 1.8‐L culture supernatant (Table 1, Fig. S1A). Next‐generation sequencing (NGS) revealed a predicted ORF of 67 013 base pairs (bp) (Table S1). The peptide sequence of WT enzyme obtained with LC‐MS was utilized as a probe, and the enzyme gene (*lpls‐pld*) was identified in the predicted ORF database as 1005 bp encoding a 334‐amino acid (aa)‐long protein (Fig. 2). A possible ribosome‐binding site (atga) was identified 40 nucleotides upstream of the start codon, gtg. Three possible terminator regions (ggccatggcg, ccggacgaggtccgg, and ggtccgggcc) were found 6–39 nucleotides downstream of the stop codon. Three possible promoter regions were found ~ 10–40 nucleotides upstream of the start codon: −35, atgtct, tggtcc, and gtgcta; −10, taaagt and gatgat. Based on SignalP prediction, the N‐terminal sequence of the active form of the enzyme starts at Thr28 of the deduced aa sequence, indicating that the preceding 27‐aa residues represent a Sec signal peptide sequence containing a signal peptidase recognition site (Ala‐Xxx‐Ala) that is required for secretion (Fig. 2). The molecular mass of the gene product (i.e., the 307‐aa protein) without the signal sequence is calculated to be 33 452 Da in agreement with that of the purified enzyme estimated by SDS/PAGE. The isoelectric point (pI) of the mature enzyme was calculated as 5.55 using genetyx‐mac version 16.0.8. SOSUI system analysis 16 indicated that LyPls‐PLD may be a membrane protein with a single transmembrane helix (^218^K to L^240^) and an average hydrophobicity value (−0.227).

**Table 1:** Purification of LyPls‐PLD from *Thermocrispum* sp. RD004668

Purification step	Total activity^a^ (U)	Total protein (mg)	Specific activity (U·mg^−1^ protein)	Yield (%)	Purification (fold)
84‐h culture supernatant	104	588	0.178	100	1.0
85% (NH_4_)_2_SO_4_ ppt	72.8	255	0.285	69.7	1.6
Giga Cap Q‐650M	29.4	92.5	0.317	28.1	1.8
PPG‐600M	13.4	6.95	1.93	12.8	10.9
RESOURCE Q	12.0	2.09	5.72	11.4	32.2
Superdex 200	7.03	0.778	9.04	6.7	50.9
RESOURCE ISO	2.69	0.157	17.2	2.6	96.8
Mono Q	1.55	3.07 × 10^−2^	50.6	1.5	285

a LyPls‐PLD activity was assayed at 37 °C for 10 min using the reaction mixture containing 80 mm Tris/HCl (pH 8.4), 2 mm CaCl_2_, and 0.4 mm LyPlsCho.

### Expression and purification of rLyPls‐PLD

High‐efficiency production of the recombinant enzyme (rLyPls‐PLD) was successfully achieved in *E. coli* BL21 (DE3) and Shuffle T7 cells transformed with pET24a/*lpls‐pld mat*_his as well as pET24a/*lpls‐pld mat*. High specific activity (345 U·mg^−1^ protein) and a large amount (~ 1 mg protein) of pure rLyPls‐PLD were purified to electrophoretic homogeneity from 200 mL Shuffle T7 *E. coli* cells culture carrying pET24a/*lpls‐pld mat*_his (Fig. S1B and Table 2). Further high‐efficiency extracellular production of rLyPls‐PLD was successfully achieved in *S. lividans* cells transformed with an expression vector pUC702 15 (data not shown).

**Table 2:** Purification of rLyPls‐PLD produced by *E. coli* transformants

Purification step	Total activity^a^ (U)	Total protein (mg)	Specific activity (U·mg^−1^ protein)	Yield (%)	Purification (fold)
cfe	1318	71.6	18.4	100	1.0
HisTrap HP	340	0.984	345	25.8	18.8

a rLyPls‐PLD activity for LyPlsCho was assayed under standard assay condition II (50 °C, pH 8.0, 1‐min reaction).

### Characterization of rLyPls‐PLD

**Figure 3:**
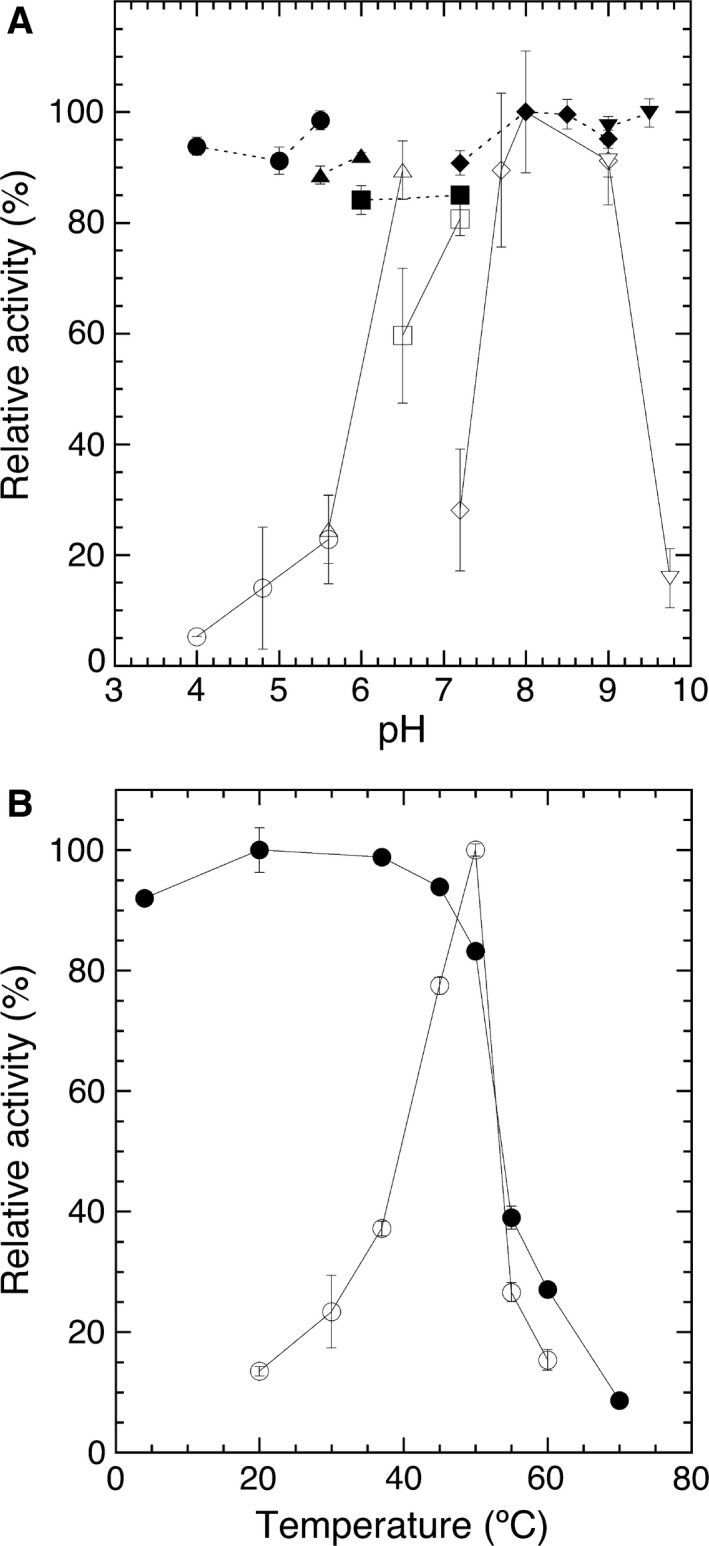
Effects of pH (A) and temperature (B) on the activity (open symbols) and stability (closed symbols) of purified rLyPls‐PLD. (A) Enzyme activity was assayed at 37 °C under the standard conditions in 80 mm of each buffer: sodium acetate (pH 4.0–5.6; open circles), MES‐NaOH (pH 5.6, 6.5; open triangles), BisTris/HCl (pH 6.5, 7.2; open squares), Tris/HCl (pH 7.2–9.0; open diamonds), and glycine‐NaOH (pH 9.0–9.75; open inverted triangles). To determine pH stability, the enzyme sample was incubated at 4 °C for 4 h in 50 mm of each buffer: sodium acetate (pH 4.1–5.5; closed circles), MES‐NaOH (pH 5.5, 6.0; closed triangles), BisTris/HCl (pH 6.0, 7.2; closed squares), Tris/HCl (pH 7.2–9.0; closed diamonds), and glycine‐NaOH (pH 9.0, 9.5; closed inverted triangles). Residual activity was assayed under standard assay condition II (50 °C, pH 8.0). (B) Enzyme activity (open circles) was assayed at each temperature under standard assay condition II. To determine thermal stability, the enzyme sample was incubated at each temperature for 60 min in 20 mm Tris/HCl buffer (pH 8.0), and residual activity (closed circles) was assayed under standard assay condition II. Data are the means of experiments performed in triplicate. Error bars represent the standard deviations.

**Figure 4:**
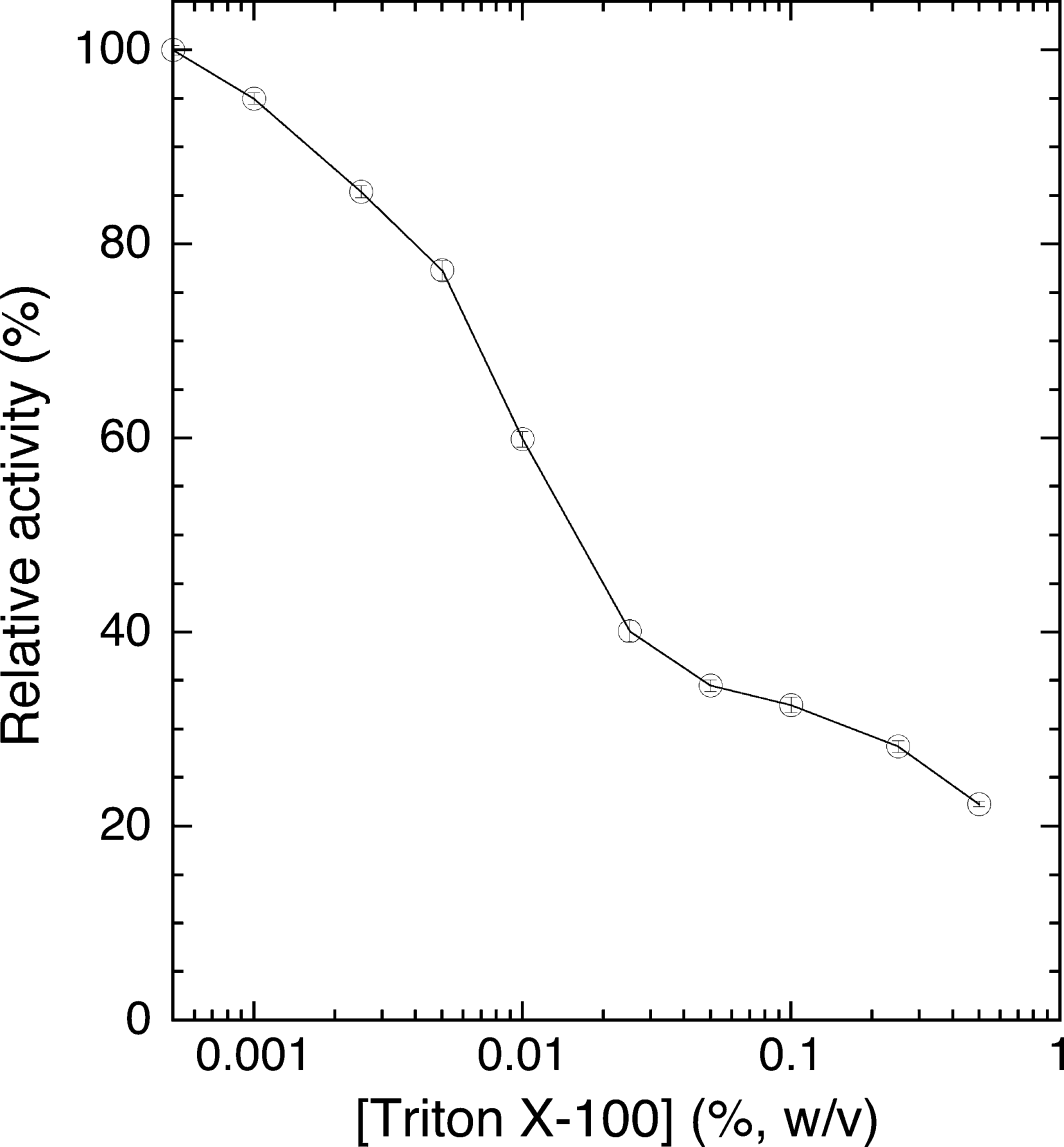
Effect of Triton X‐100 concentration on enzyme activity. LyPls‐PLD activity was assayed under standard assay condition I containing each Triton X‐100 concentration. Data shown represent the mean ± standard deviation (*n* = 3).

**Figure 5:**
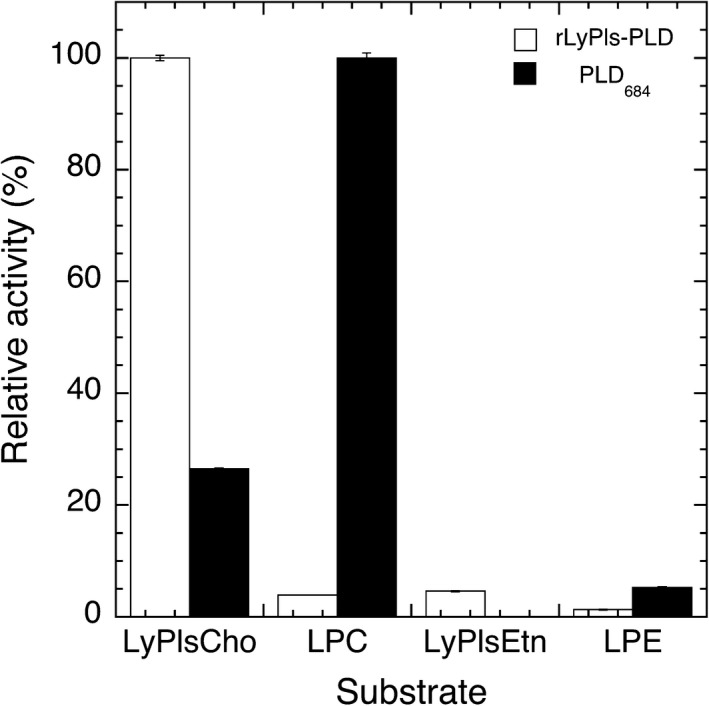
Substrate specificities of rLyPls‐PLD (open bar) and PLD_684_ (closed bar). rLyPls‐PLD and PLD_684_ activities 15 were assayed under standard assay condition I. Data shown represent the mean ± standard deviation (*n* = 3).

The highest enzyme activity for LyPlsCho hydrolysis was found at 50 °C and pH 8.0 (Fig. 3). Enzyme activity was maintained between pH 4.1 and 9.75 at 4 °C for 4 h. The enzyme was stable between 4 and 37 °C for 60 min at pH 8.0. LyPls‐PLD was activated in the presence of 0.5–10 mm CaCl_2_ and 25 μm–5 mm AlCl_3_ but was inhibited by 2 mm MgCl_2_, EDTA (Table 3), and > 5 mm Al(III) (data not shown). Dithiothreitol (DTT), 2‐mercaptoethanol (2ME), iodoacetamide (IAA), and phenylmethylsulfonyl fluoride (PMSF) had no effect on enzyme activity (data not shown). Although there are two cysteine residues in LyPls‐PLD, no disulfide bond formed judging from the effects of the reducing reagents (DTT and IAA). Enzyme activity decreased with a higher concentration of Triton X‐100 in the reaction mixture (Fig. 4). The enzyme activity in the absence of Triton X‐100 was as high as that in the presence of 5 ppm Triton X‐100. LyPls‐PLD hydrolyzed liposomal LyPlsCho composed of 1 mm 1‐palmitoyl‐2‐oleoyl‐*sn*‐glycero‐3‐phosphocholine (POPC)/0.4 mm LyPlsCho with modest activity (51.6% activity found using micellar LyPlsCho substrate). l‐α‐Lysophosphatidylcholine (LPC), l‐α‐lysophosphatidylethanolamine (LPE), and 1‐stearoyl‐2‐hydroxy‐*sn*‐glycero‐3‐phosphate (LPA) inhibited enzyme activity, but GPC and CDP‐choline did not (Table 4). The enzyme hydrolyzed highly specific for LyPlsCho; LPC, LyPlsEtn, and LPE were < 5% activity compared with that found using LyPlsCho (Fig. 5). However, POPC, 1‐palmitoyl‐2‐oleoyl‐*sn*‐glycero‐3‐phosphoethanolamine (POPE), PlsCho, PlsEtn, sphingomyelin (SM), and GPC were not hydrolyzed in any Triton X‐100 concentration tested (data not shown).

**Table 3:** Effect of metal ions on rLyPls‐PLD activity

Chemicals	Relative activity (%)^a^
None	37.3 ± 0.6
CaCl_2_	100 ± 0.7
MgCl_2_	5.4 ± 0.3
NaCl	38.1 ± 0.6
KCl	37.9 ± 0.5
AlCl_3_	84.5 ± 0.7
EDTA	0

a rLyPls‐PLD activity was assayed under standard assay condition I. The relative activity was determined by defining the activity without chemicals as 100%. Data represent the means and standard deviations of experiments performed in triplicate.

**Table 4:** Effect of substrate analogs on rLyPls‐PLD activity

Substrate analogs	Relative activity (%)^a^
None	100 ± 0.6
LPC	60.7 ± 0.5
LPE	50.9 ± 0.4
LPA	89.8 ± 0.3
GPC	99.0 ± 0.3
CDP‐choline	99.4 ± 0.5

a rLyPls‐PLD activity was assayed under standard assay condition I containing 4 mm of each substrate analog. The relative activity was determined by defining the activity without substrate analog as 100%. Data represent the means and standard deviations of experiments performed in triplicate.

### Comparative sequence analysis of LyPls‐PLD

**Figure 6:**
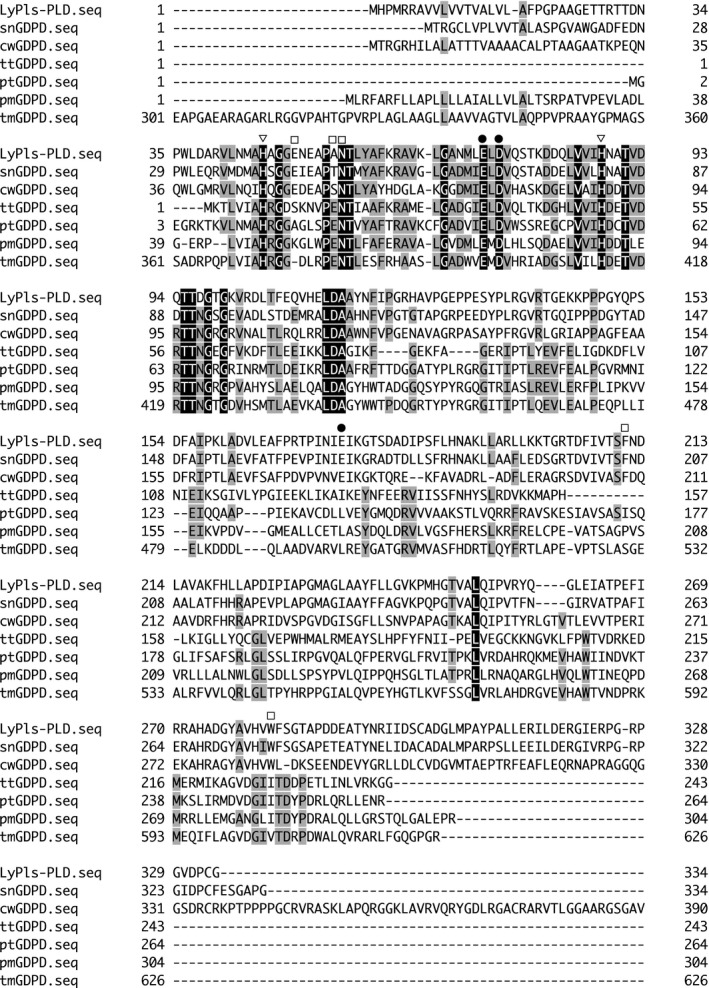
Multiple‐sequence alignment of LyPls‐PLD and GDPDs. The alignment was performed using genetyx‐mac ver. 17.0.8 (GENETYX Corp., Tokyo, Japan). snGDPD, GDPD from *Stackebrandtia nassauensis* strain DSM 44728 (UniprotKB accession number D3Q1U5); cwGDPD, GDPD from *Conexibacter woesei* strain DSM 14684 (D3F5R1); ttGDPD, GDPD from *Caldanaerobacter subterraneus subsp. tengcongensis* (*Thermoanaerobacter tengcongensis*) strain DSM 15242 (Q8RB32); ptGDPD, GDPD from *Pelotomaculum thermopropionicum* strain DSM 13744 (A5D3P2); pmGDPD, GDPD from *Pseudomonas mendocina* strain ymp (A4XQ14); tmGDPD, GDPD from *Thermaerobacter marianensis* strain ATCC 700841 (E6SMM5). Highly conserved amino acids are shaded in black and gray for each protein. Symbols: open triangles, putative catalytic residues; closed circles, putative metal ion‐binding sites; open squares, predictive substrate recognition residues.

**Figure 7:**
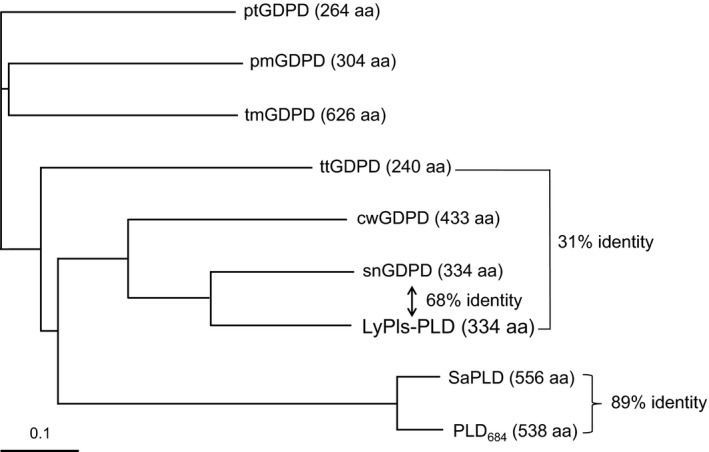
Tree view exhibiting amino acid sequence similarity of LyPls‐PLD with the other GDPDs and PLDs. The tree was constructed using ClustalW. The 0.1 scale represents a genetic unit reflecting 10% of the amino acid substitutions. The phylogenetic tree drawn using TreeView includes the following: ptGDPD, pmGDPD, tmGDPD, ttGDPD, cwGDPD, snGDPD, SaPLD of *Streptomyces antibioticus* (UniprotKB accession number Q53728), and PLD_684_ (DDBJ accession number AB771745).

A homology search performed using the BLAST algorithm demonstrated that the aa sequence of the mature LyPls‐PLD shared 68% and 31% identity with GDPDs from *Stackebrandtia nassauensis* (snGDPD; UniProt accession no. D3Q1U5) 17 and *Thermoanaerobacter tengcongensis* (ttGDPD; UniProt accession no. Q8RB32) 18, and showed high similarity with other GDPDs as well (Fig. 6). However, most of them remain to be biochemically and functionally characterized. The deduced aa sequence of LyPls‐PLD shared no similarity to those of PLDs and contained no HKD motifs conserved in many PLDs 19. A distance‐based phylogenetic analysis revealed that LyPls‐PLD belonged to a member of the GDPD family and was clearly separated from a group containing PLD (Fig. 7). Intriguingly, this suggests that GDPD might be an ancestor protein of PLD. Moreover, LyPls‐PLD was clearly separated from at a group containing ptGDPD, pmGDPD, and tmGDPD. Pfam analysis 20 showed that the N terminus (residues 19–271) of LyPls‐PLD was assigned as a GDPD family domain and shared structural similarity with ttGDPD (Protein Data Bank code: 2PZ0) and GDPD from *Parabacteroides distasonis* (pdGDPD; Protein Data Bank code: 3NO3).

### Homology modeling analysis and molecular docking

**Figure 8:**
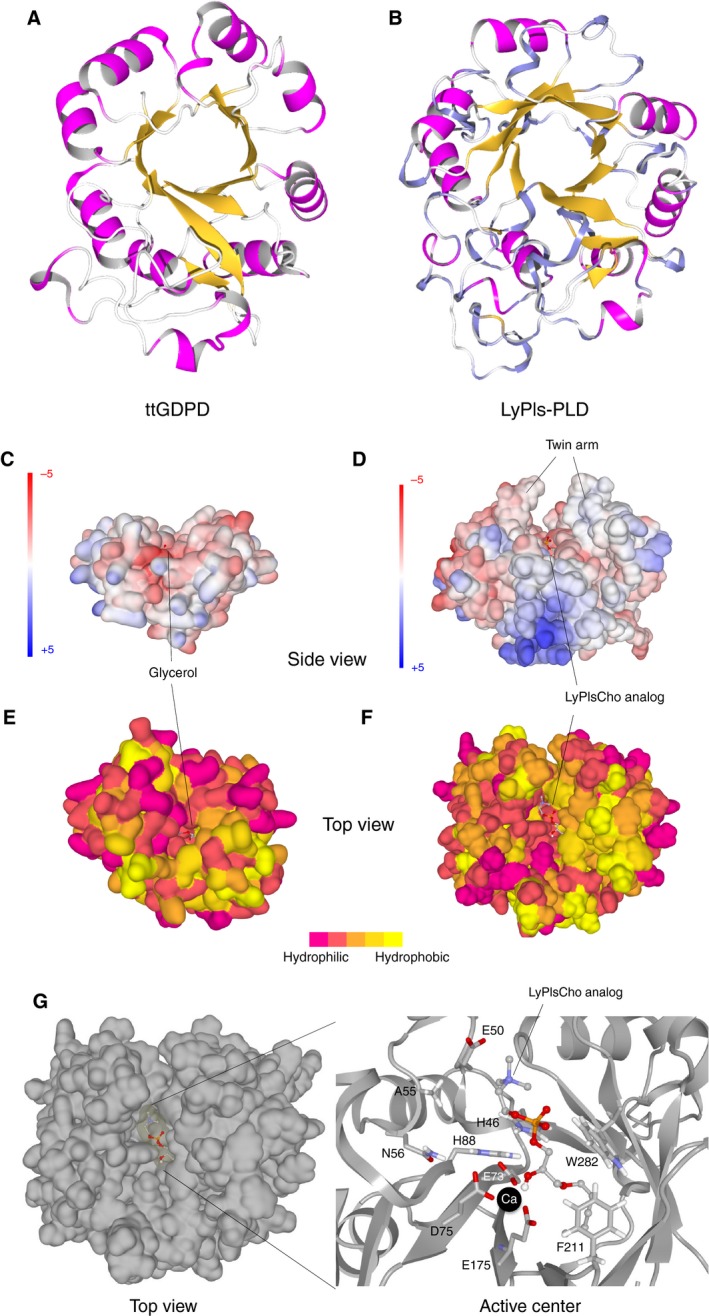
Ribbon representation of ttGDPD structure (panel A, Protein Data Bank code: 2PZ0) and homology‐modeled structure of LyPls‐PLD (B). The structures are represented in ribbon models from the same viewpoint. The secondary structural elements are shown in magenta (α‐helix) and yellow (β‐strand). Surface representations of ttGDPD (C, E) and LyPls‐PLD (D, F, G). Panels C and D, the surface models are colored according to the electrostatic potential, from −5 (red, negative charge) to +5 (blue, positive charge). Panels E and F, the surface models are colored according to the hydrophobicity, from magenta (hydrophilic) to yellow (hydrophobic) in five steps. Panel G, putative active site of LyPls‐PLD. Amino acid residues are depicted as stick model. The active site computationally docked with C3‐alkenyl‐LyPlsCho of the substrate analog depicted as a stick‐and‐ball model. The putative Ca^2+^‐binding position is shown as a black ball.

VERIFY3D showed that the modeled structure exhibited 116.6 of 3D–1D total score (i.e., high modeling quality), suggesting that it is sufficient for comparative structural discussion. The modeled structure of LyPls‐PLD showed high similarity to the crystal structures of GDPDs such as Protein Data Bank code: 3L12, 4R7O, 2OOG, 3QVQ, and 3KS6 and adopted a TIM barrel fold (Fig. 8). ttGDPD is one of the few GDPDs that have been well studied and characterized. Thus, we compared the structural feature of LyPls‐PLD and ttGDPD. Twin arm structure and deep hydrophobic pocket were observed in LyPls‐PLD, but not in ttGDPD (Fig. 8C,D). The H46, E73, D75, H88, E175, F211, and W282 residues in LyPls‐PLD were highly conserved with ttGDPD (Figs 6 and 8G). We speculated that E73, D75, and E175 are probably calciumion (Ca^2+^)‐binding sites. The docking simulation indicated that the calculated free energy of LyPlsCho analog binding was −4.2 kJ·mol^−1^. It also suggested that H46 and H88 of the putative catalytic residues were located near phosphate group of the substrate analog, and E50, A55, and N56 were observed near the head group of the substrate analog (Fig. 8G).

### Mutational analysis

Except for A55N/D, N56D/Q, E175A, and F211A, we were unable to successfully express other mutant enzymes. The E175A variant was inactive, suggesting that the residue may be important for enzyme activity. For LyPlsCho, the enzyme activities of mutants A55N/D, N56D/Q, and F211A were remarkably decreased compared with that of WT enzyme, especially N56D and F211A (Table 5). For LyPlsEtn, the enzyme activities of A55N/D and N56D variants were also markedly declined compared with that of WT enzyme; however, in the N56Q and F211A variants, there were slight decrease in the activities. Interestingly, the F211A variant exhibited higher activity toward PlsEtn than WT enzyme, but the enzyme activity of the N56Q variant markedly declined. Also, the other mutations had almost no effect on PlsEtn hydrolysis.

**Table 5:** Hydrolytic activities of WT enzyme and mutants with LyPls and PlsEtn

Variants	Relative activity (%)^a^		
	LyPlsCho	LyPlsEtn	PlsEtn
WT	100	6.48	0.971
A55N	51.6	2.94	0.784
A55D	47.0	3.78	1.01
N56D	8.38	1.83	0.811
N56Q	55.2	5.42	0.410
F211A	20.0	5.53	1.24

WT = wild‐type enzyme.

a The enzyme activity was assayed under standard assay condition II. The relative activity was determined by defining the activity of WT enzyme as 100%.

### Enzyme kinetics

Kinetic parameters were summarized in Table 6. WT enzyme exhibited much higher affinity toward for LyPlsCho and catalytic efficiency for LyPlsCho hydrolysis than LyPlsEtn. The *V*_max_ value was 123 μmol·min^−1^·mg^−1^ protein (62.5 μm·min^−1^). The apparent *K*_m_ and *k*_cat_/*K*_m_ values were 13.2 μm and 5345 s^−1^·mm ^−1^, respectively (Fig. S2). The catalytic efficiency (*k*_cat_/*K*_m_) of F211A mutant for LyPlsCho was remarkably decreased as compared with LyPlsEtn.

**Table 6:** Steady‐kinetic parameters of WT enzyme and F211A mutant

	*k*_cat_ (s^−1^)		*K*_m_ (mm)		*k*_cat_/*K*_m_ (s^−1^·mm ^−1^)	
	LysPlsCho	LysPlsEtn	LysPlsCho	LysPlsEtn	LysPlsCho	LysPlsEtn
WT	70.6	4.51	1.32 × 10^−2^	0.456	5345	9.9
F211A	24.8	2.88	0.112	0.281	221	10.3

WT = wild‐type enzyme.

The enzyme activity was assayed under standard assay condition II.

## Discussion

**Figure 9:**
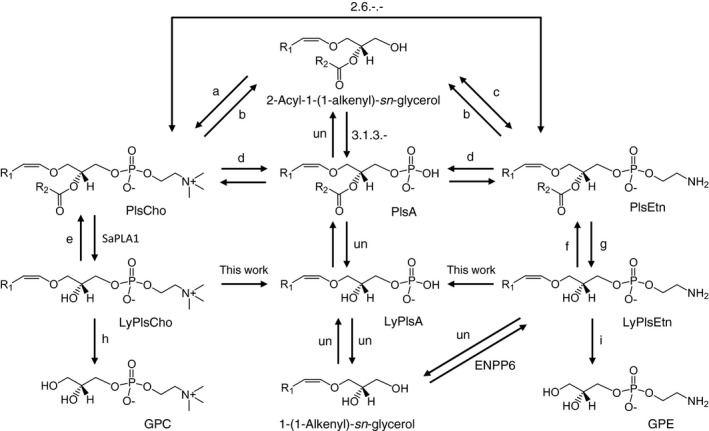
Predicted pathway and enzymes concerned with Pls metabolism. a, 1‐alkenyl‐2‐acylglycerol choline phosphotransferase (EC 2.7.8.22); b, PLC (EC 3.1.4.3); c, ethanolamine phosphotransferase (EC 2.7.8.1); d, PLD (EC 3.1.4.4); e, 1‐alkenylglycerophosphocholine *O‐*acyltransferase (EC 2.3.1.25); f, 1‐alkenylglycerophosphoethanolamine *O‐*acyltransferase (EC 2.3.1.23); g, PLA_2_ (EC 3.1.1.4); h, alkenylglycerophosphocholine hydrolase (EC 3.3.2.2); i, alkenylglycerophosphoethanolamine hydrolase (EC 3.3.2.5); ENPP6, ectonucleotide pyrophosphatase/phosphodiesterase family member 6 (EC 3.1.4.URLHYPHEN;) 56; SaPLA_1_, PLA_1_ from *S. albidoflavus* NA297; PlsA, 2‐acyl‐1‐(1‐alkenyl)‐*sn*‐glycero‐3‐phosphate; LyPlsA, 1‐(1‐alkenyl)‐*sn*‐glycero‐3‐phosphate; un, unknown.

There is limited information on Pls metabolic pathways and its key enzymes in living organisms. We identified and characterized a novel LyPls‐PLD involved in Pls metabolism, in particular, in microorganisms (Fig. 9). Moreover, the ORF of *lpls‐pld* was identified with NGS, and the peptide was sequenced with LC‐MS. We also efficiently produced rLyPls‐PLD using *E. coli*.

The putative gtg translational start codon of LyPls‐PLD was similar to ttg in *Streptoverticillium cinnamoneum* PLD (StvPLD) 21. The signal peptide of LyPls‐PLD had a typical Sec signal sequence, suggesting that it should be secreted via the secretory pathway (Sec‐system secretion) 22. Most *Streptomyces* phospholipases are secreted via the Sec‐system pathway 15, 23, 24, 25, 26 except for PLC 23 and C‐type enzymes such as glycerophosphocholine cholinephosphodiesterase (GPC‐CP) 27 and glycerophosphoethanolamine ethanolaminephosphodiesterase (GPE‐EP) 28 that are secreted via a Tat‐system pathway. The deduced aa sequence of LyPls‐PLD showed high similarity to those of other bacterial GDPDs such as snGDPD 17 and ttGDPD, but no similarity to those of known PLDs. The catalytic reaction of LyPls‐PLD is identical to those of GDPD and PLD in terms of hydrolysis of the same phosphoester bond; however, the substrate specificity differs completely from those of GDPD and PLD. In general, GDPDs display broad specificity for glycerophosphodiesters; GPC, GPE, glycerophosphoglycerol, and bis(glycerophosphoglycerol) 18, 29. Conversely, LyPls‐PLD exhibited no activity toward GPC, DAGPLs, or Pls but high specificity to LyPlsCho. Moreover, the phylogenetic analysis revealed that LyPls‐PLD belongs to GDPD superfamily because of clear separation from a group containing one other GDPD group as well as PLD. It also indicates that the GDPD gene could be an ancestral gene of PLD. According to Pfam, the GDPD family is a member of the clan PLC (CL0384), which contains two GDPD families and two phosphoinositide (PI)‐PLC families. The GDPD family appears to have weak similarity to mammalian PI‐PLCs, suggesting that the family may adopt a TIM barrel fold (PF00388).

The enzyme activity of LyPls‐PLD was maximal at pH 8.0, resembling that of *Streptomyces chromofuscus* PLD (ScPLD; pH 7.5–8.5) 30 or ttGDPD (pH 9.0) 31 and *E. coli* GDPD (pH 9.0) 29, but not the other *Streptomyces* PLDs (pH 5.0–6.0) 23, 24, 32. The predicted pI 5.55 of LyPls‐PLD was much lower than pI 8 of PLD from *Streptomyces racemochromogenes* strain 10‐3 33 but similar to those of ScPLD 30, plant PLDs such as *Arabidopsis thaliana* 34, and *Vitis vinifera* 35, which are 5.1, 5.6, and 6.27, respectively. We hypothesized that the higher maximal pH of LyPls‐PLD is perhaps required for Ca^2+^ to act as the electrophile (Fig. S3). The observed maximum temperature of enzyme activity (50 °C) is almost the same as those of other *Streptomyces* PLDs (50–60 °C) 23, 24, 32. It is lower than *Streptomyces tendae* PLD (70 °C) 32 and *Streptomyces olivochromogenes* PLD (75 °C) 32 but matches the optimum growth temperature (45 °C) of strain RD004668. LyPls‐PLD was stable over a broad pH range (pH 4–10) at 4 °C for 4 h and at 4–37 °C (pH 8.0) for 1 h; however, incubation at 70 °C for 1 h (pH 8.0) decreased relative activity to < 10%, indicating that the enzyme is somewhat heat unstable compared with other PLDs. As for GDPD, there is no published information about these characteristics.

Liang *et al*. proposed that ttGDPD binds substrate via the coordination effect of a metal ion, and a possible role for the ion in the catalytic reaction would be acting as an electrophile to stabilize the reaction intermediate 18. This is similar to the coordination effect of metal ion on PI‐PLC. Almost all *Streptomyces* PLDs exhibit Ca^2+^‐independent activity 19; however, some PLDs such as from *S. racemochromogenes* 33, *S. olivochromogenes* 36, and *S. tendae* 32 are Ca^2+^‐dependent enzymes, and the iron‐containing enzyme ScPLD is activated by Ca^2+^ 19, 37. Additionally, both Ca^2+^‐dependent and ‐independent PLDs have been found in mammals, yeasts, bacteria, and nearly all plants; these PLDs also require a micro‐ to millimolar Ca^2+^ concentration to stimulate activity 38. Because LyPls‐PLD is a Ca^2+^‐dependent enzyme, the catalytic mechanism of LyPls‐PLD might be similar to that of Ca^2+^‐dependent PLD or GDPDs. In fact, H46 and H88 in LyPls‐PLD were highly conserved with those in the catalytic residues of GDPDs as well as PLD. The X‐ray crystal structure analysis of ttGDPD revealed that E44, D46, E119, two water molecules, and the OH group of glycerol compose an octahedral arrangement that exhibits tetragonal bipyramidal coordination with a glycerol molecule binding at one Ca^2+^ ion; however, native ttGDPD activity requires Mg^2+^ but not Ca^2+^ as a cofactor 18. It is well known that hard acids such as Ca^2+^ and Mg^2+^ form a complex with hard bases such as water molecules, OH^−^, COO^−^, phosphate (${\text{PO}}_{3}^{4-}$), and RNH_2_. Thus, most phosphodiesterases such as acid phosphatase from *Francisella tularensis* (Protein Data Bank code: 2D1G) 39 and GPC‐CP 27 and GPE‐EP 28 from *Streptomyces sanglieri* appear to utilize Ca^2+^ to activate the serine OH group and form a covalent serine‐phosphoryl intermediate. The inhibitor test demonstrated that active serine and cysteine residues would not affect LyPls‐PLD activity. Interestingly, LyPls‐PLD was activated by Ca^2+^ and inhibited by Mg^2+^; however, ttGDPD exhibits high activity in the presence of Mg^2+^ but lower activity in the presence of Ca^2+^ 18. The reason underlying the discrepant metal ion‐dependency between ttGDPD and LyPls‐PLD remains unclear. With regard to LyPls‐PLD, calcium ion may play a key role in stabilizing phosphate group of the substrate and the reaction product, lysoplasmenic acid (LyPlsA; 1‐(1‐alkenyl)‐*sn*‐glycero‐3‐phosphate) as well as role as electrophile (Fig. S3). The LyPls‐PLD hydrolytic activity was also enhanced ~ 2‐fold in the presence of 0.1–1 mm AlCl_3._ The effect of Al(III) on LyPls‐PLD activity seems be the intrinsic property. Likewise, PLD hydrolytic activity from *Actinomadura* sp. No. 362 was also stimulated ~ 1.4‐fold by 1 mm AlCl_3._ 40. Ogino *et al*. reported that the transphosphatidylation activity for PC and PE of StvPLD was enhanced by Al(III) (~ 2.5‐fold); however, the hydrolytic activity was unaffected with up to 5 mm Al(III) and was completely inhibited by > 5 mm Al(III) 21. We hypothesize that the physical condition of the substrate micelles (e.g., size and form) was changed with the concentration of Al(III), thus affecting activity. We previously reported a similar result upon enhancement of sphingomyelinase activity by Mg^2+^ ions 41.

We also assessed the effect of Triton X‐100 on LyPls‐PLD activity. The hydrolytic activity of *Streptomyces* phospholipases are generally stimulated by Triton X‐100 25, 26, 32, 42, 43, 44 because their substrate recognition mechanisms appear to depend on substrate form such as emulsified substrate or mixed micelle substrate with Triton X‐100 15. In contrast, LyPls‐PLD activity declined with increasing Triton X‐100 concentration in the reaction mixture. We recently reported that the substrate recognition mechanism of PLD_684_ depended on substrate forms and preferred mixed micelle substrates to liposomal substrates 15. Likewise, LyPls‐PLD preferred the micelle‐formed LyPlsCho substrate to the liposomal LyPlsCho composed of 1 mm POPC/0.4 mm LyPlsCho with modest activity (51.6% activity found using micellar LyPlsCho). This indicates that LyPls‐PLD may prefer unimolecular micelle or liposomal substrate but not Triton X‐100/LyPlsCho‐mixed micelle.

The structure modeling analysis suggested that LyPls‐PLD would be similar to the crystal structure of ttGDPD as well as other GDPDs but not those of bacterial PLDs 19, 45. Also, the model structure of LyPls‐PLD appeared to adopt a TIM barrel fold. Surprisingly, conjugated polyketone reductase C2 (Protein Data Bank code: 4H8N) from *Candida parapsilosis* (NADPH‐dependent ketopantoyl lactone reductase) also adopts a TIM barrel fold, and its active site interacts with the two phosphate groups of NADPH 46. Thus, the TIM barrel fold could play a key role in the interaction with the phosphate group. Shi *et al*. 18 discussed the catalytic mechanism of ttGDPD using its crystal structure. Based on their findings, we speculated that the catalytic mechanism of LyPls‐PLD would be similar to those of ttGDPD and PLD 47. Namely, LyPls‐PLD would bind the substrate via the coordination effect of Ca^2+^, and two histidines (H46 and H88) certainly play a role as acid–base catalyst like in ttGDPD and PLD (Fig. S3). With regard to the catalytic residue, two histidines were conserved between LyPls‐PLD and ttGDPD, as well as in other GDPDs and PLDs. Interestingly, GPC‐CP and GPE‐EP (i.e., a PLC‐like enzyme), which cleave GPC or GPE into glycerol and phosphocholine or phosphoethanolamine, utilize two histidines to bind the substrate's phosphate 28. However, the substrate specificity of LyPls‐PLD completely differs from those of GDPD and PLD.

Mutational analysis demonstrated that A55 and N56 might be involved in the head group recognition. The N56D mutant, which changes from amide group of Asn to the negative charge of carboxyl group of Asp residue at reaction pH 8, unexpectedly exhibited a remarkable decrease of LyPlsCho hydrolysis activity but had a minimal effect on PlsEtn hydrolysis activity. However, the same mutation had a small effect on LyPlsEtn hydrolysis activity compared with LyPlsCho. Most interestingly, the N56Q variant, in spite of locating distant from *sn*‐2 acyl chain of PlsEtn, exhibited ~ 40% activity for PlsEtn compared with the activity of the WT enzyme, whereas the other variants, except for F211A, had a modest and almost no effect on PlsEtn hydrolysis. Based on these results, we reasoned that replacement of Asn56 with Gln is a mutation that permits LyPlsEtn hydrolysis but not PlsEtn, while a longer chain might interrupt LyPlsCho binding due to a bulky Cho rather than an Etn head group. Moreover, the kinetic analysis demonstrated that the affinity of LyPlsCho to the enzyme markedly decreased in F211A mutant due to the replacement of Phe, which has a bulky and hydrophobic side chain, by Ala with a small methyl group. This suggests that the Cho head group might interact hydrophobically with Phe group of F211 when the enzyme incorporates the substrate. However, F211 and W282 were located in close proximity to *sn*‐1 ether bond and its alkyl chain of the substrate analog, but not near the Cho head group. Thus, we considered that F211 and W282 residues could interact with the *sn*‐1 ether bond and its alkyl chain of LyPls substrate. Further investigation into the protein's structure is required to elucidate the mechanisms responsible for LyPls‐PLD substrate recognition. Crystal structure determination and more detailed mutation analyses are currently in progress.

The inhibition study results suggested that compounds such as GPC and CDP‐choline with no acyl chain and ether bond were not LyPls‐PLD substrates and were unable to enter enzyme's active center. Compared with LPC and LPE, the inhibitory effect of LPA was weak; however, LPC and LPE can be LyPls‐PLD substrates. Additionally, LyPls‐PLD preferred the Cho head group over Etn of lysophospholipids as well as LyPls. Wu *et al*. 1 reported that 50% of LyPlsase activity was inhibited with 50 μm LPA. As for LyPls‐PLD inhibition, a concentration of 4 mm analog (10‐fold higher concentration toward LyPlsCho substrate) was required to inhibit enzyme activity (Table 4). Substrate specificity profiling of LyPls‐PLD showed that the enzyme recognizes a vinyl ether bond at the *sn*‐1 position and the head group (Fig. 5, Tables 5 and 6). Taken together, we concluded that LyPls‐PLD would preferentially recognize the *sn*‐1 vinyl ether bond and then the head group. Yet, LyPls‐PLD likely prefers vinyl ether bond over acyl ester bond at the *sn*‐1 position.

Finally, we concluded that LyPls‐PLD is a novel LyPlsCho‐specific enzyme that is clearly different from any known PLD as well as GDPD; however, the catalytic mechanism of LyPls‐PLD seems similar to that of ttGDPD rather than PLD (Fig. S3). The identification of LyPls‐PLD reveals a new pathway for Pls metabolism, in particular, in microorganisms. We showed that LyPls‐PLD is obviously different from PLD and the phosphodiesterase families containing GDPD with regard to the catalytic function, substrate recognition mechanism, and biochemical roles. More detailed mutation analyses are currently in progress to elucidate the substrate recognition mechanism of LyPls‐PLD. Further advances in this area could lead to the development of diagnostic reagent kits for early stages of diseases such as Alzheimer's disease and arteriosclerosis.

## Materials and methods

### Materials

1‐*O‐*1′‐(Z)‐octadecenyl‐2‐hydroxy‐*sn*‐glycero‐3‐phosphocholine (LyPlsCho), 1‐(1Z‐octadecenyl)‐2‐arachidonoyl‐*sn*‐glycero‐3‐phosphocholine (PlsCho), LPC, LPA, POPC, 1‐*O‐*1′‐(Z)‐octadecenyl‐2‐hydroxy‐*sn*‐glycero‐3‐phosphoethanolamine (LyPlsEtn), 1‐(1Z‐octadecenyl)‐2‐arachidonoyl‐*sn*‐glycero‐3‐phosphoethanolamine (PlsEtn), and POPE were purchased from Avanti Polar Lipids, Inc. (Alabaster, AL, USA). LPE was purchased from Doosan Serdary Research Laboratories (Toronto, ON, Canada). SM and Etn hydrochloride were purchased from Sigma‐Aldrich Japan Co., LLC (Tokyo, Japan). GPC was purchased from Bachem AG (Torrance, CA, USA). CDP‐choline and Cho hydrochloride were purchased from Wako Pure Chemical Industries Ltd. (Osaka, Japan). Bacto‐peptone and Bacto‐malt extract were purchased from Becton, Dickinson and Company (Franklin Lakes, NJ, USA). Toyopearl Giga Cap Q‐650M and Toyopearl PPG‐600M were purchased from Tosoh (Tokyo, Japan). RESOURCE Q, RESOURCE ISO, Mono Q, Superdex 200 10/300 GL, and HisTrap HP columns were purchased from GE Healthcare Japan (Tokyo, Japan). Choline oxidase (COD) from *Arthrobacter globiformis* was from Asahi Kasei Pharma (Tokyo, Japan). Peroxidase (POD) and yeast extract BSP‐B were purchased from Oriental Yeast Co., Ltd. (Tokyo, Japan). 4‐Aminoantipyrine (4‐AA) was purchased from Nacalai Tesque Inc. (Kyoto, Japan). *N,N*‐Bis(4‐sulfobutyl)‐3‐methylaniline, disodium salt (TODB) was purchased from Dojindo Laboratories (Kumamoto, Japan). His6‐tagged recombinant amine oxidase (rSrAOX) from *Syncephalastrum racemosum* was produced using pET24a(+)/*E. coli* and purified using affinity column chromatography with HisTrap HP 48. All other chemicals were of the highest or analytical grade.

### Bacterial strains, plasmids, and culture conditions

Approximately 200 actinomycetes strains were obtained from NBRC (the NITE Biological Resource Center, Chiba, Japan), and LyPls‐PLD producers were screened with enzyme activity assays. Strain RD004668 (RD4668) showed the highest LyPls‐PLD activity in 5 mL cultivation and was selected for further investigation. Strain RD4668 from excrement of Kanagawa, Japan was identified as *Thermocrispum* sp., a near relative of *Thermocrispum municipal,* based on morphological, physiological, biochemical characterizations, and 16S rDNA sequence analysis. The 16S rDNA sequence of strain RD4668 was deposited in the DDBJ database under accession number AB873024. Strain RD4668 was deposited as NITE BP‐01628 in the NITE Patent Microorganisms Depositary (NPMD; Chiba, Japan). The optimum growth temperature of strain RD4668 is 45 °C. Strain RD4668 was incubated in 5 mL ISP2 medium (1% malt extract, 0.4% yeast extract, 0.4% glucose, pH 7.3) supplemented with 0.41 mm Brij35 at 45 °C for 48 h with shaking (160 strokes per min). Next, 1 mL of the 48‐h culture was transferred into a 500‐mL flask containing 100 mL ISP2 medium and incubated at 45 °C for 84 h with shaking (180 rpm).

### Purification of LyPls‐PLD

All procedures were performed at 4 °C. The culture supernatant was obtained from the 2.4 L culture by centrifugation (18 800 ***g***, 10 min) after 84 h of culturing. The resulting 1.8 L culture supernatant was placed in (NH_4_)_2_SO_4_ solution at 85% saturation containing buffer A (20 mm Tris/HCl buffer, pH 8.0) for 2 h. The precipitation was collected by centrifugation (18 800 ***g***, 10 min) and suspended in buffer B (buffer A, pH 9.0), followed by dialyzing against buffer B. After removing insoluble materials by centrifugation (21 800 ***g***, 10 min), the obtained supernatant was loaded onto a Toyopearl Giga Cap Q‐650M column (2.5 × 4.0 cm) equilibrated with buffer B. After washing the column with three column volumes (3 CV), the bound proteins were eluted with a linear gradient (20 CV) of 0 to 1 m NaCl in buffer B at 2 cm·min^−1^. Ammonium sulfate was added to the active fractions to 1.5 m followed by loading onto a Toyopearl PPG‐600M column (2.5 × 4.0 cm) equilibrated with 1.5 m (NH_4_)_2_SO_4_/buffer A. After washing, the proteins were eluted with a linear gradient (15 CV) of 1.5 to 0 m (NH_4_)_2_SO_4_ in buffer A and with buffer A (10 CV) at 2 cm·min^−1^. The active fractions were pooled, and the buffer was replaced with buffer B using Vivaspin 20–10 000 MWCO (Sartorius AG, Göettingen, Germany) followed by loading onto a RESOURCE Q column (1 mL) equilibrated with buffer B. The proteins were eluted with a linear gradient (40 CV) of 0 to 0.5 m NaCl in buffer B at 6 cm·min^−1^. The active fractions were concentrated using Vivaspin followed by loading onto a Superdex 200 column (24 mL) equilibrated with 0.15 m NaCl/buffer A. The proteins were eluted with the same buffer at 37.5 cm·h^−1^. The active fractions were exchanged to 1.5 m (NH_4_)_2_SO_4_/buffer A using Vivaspin followed by loading onto a RESOURCE ISO column (1 mL) equilibrated with the same buffer. After washing, the proteins were eluted with a linear gradient (40 CV) of 1.5 to 0 m (NH_4_)_2_SO_4_ in buffer A at 6 cm·min^−1^. The buffer of the active fractions was exchanged to buffer B using Vivaspin followed by loading onto a Mono Q column (1 mL) equilibrated with the same buffer. After washing, the proteins were eluted with a linear gradient (40 CV) of 0 to 0.4 m NaCl in buffer B at 6 cm·min^−1^. Fractions exhibiting high specific LyPls‐PLD activity and running as a single band on SDS/PAGE were pooled and used in subsequent investigations.

### Enzyme activity assays

The WT enzyme activity for LyPlsCho was assayed by measuring the Cho formation rate as follows. Solutions of TODB, 4‐AA, and POD were prepared in distilled water. The standard assay mixture (50 μL) containing 80 mm Tris/HCl (pH 8.0), 0.4 mm LyPlsCho, and 2 mm CaCl_2_ was incubated at 37 °C for 5 min. The assay was started by addition of 10% (v/v) enzyme solution and further incubation at 37 °C for 10 min (standard assay condition I). A colorimetric solution (200 μL) containing 0.03% (w/v) 4‐AA, 0.02% (w/v) TODB, 0.75 U·mL^−1^ COD, 5 U·mL^−1^ POD, and 10 mm EDTA was added to the enzyme reaction mixture to stop the reaction and determine the concentration of Cho ([Cho]) released by the enzyme reaction. Absorbance at 550 nm (A_550_) based on a quinone dye generated by the coupling reaction of H_2_O_2_ with 4‐AA and TODB was measured using a Multiskan FC microplate reader (Thermo Fisher Scientific K.K., Kanagawa, Japan). The concentration of produced H_2_O_2_ ([H_2_O_2_]) was determined by measuring the absorbance at 550 nm (A_550_) using a calibration curve generated from known [H_2_O_2_]. One unit (U) of enzyme activity was defined as the amount of enzyme that released 1 μmol Cho from LyPlsCho per min. The enzyme activity for LyPlsEtn was assayed by measuring the rate of Etn formation. The concentration of Etn ([Etn]) released by the enzyme reaction with the standard assay mixture containing 0.4 mm LyPlsEtn was determined using 0.75 U·mL^−1^ rSrAOX instead of COD, and the others were determined as described above.

### NGS and ORF prediction

The chromosomal DNA of strain RD4668 was purified according to Pospiech and Neumann 49. The genome sequencing was performed using Illumina HiSeq 2000 (Illumina Inc., San Diego, CA, USA) in accordance with the manufacturer's instructions. Adaptor trimming and assembling were performed using cutadapt (https://cutadapt.readthedocs.io/en/stable/) and Velvet (http://www.ebi.ac.uk/~zerbino/velvet/), respectively. ORFs were predicted by getorf (http://emboss.bioinformatics.nl/cgi-bin/emboss/getorf). The ORF database was constructed and utilized for the proteinlynx global server database.

### Protein analyses

The protein concentration was determined with a bicinchoninic acid protein assay reagent kit (Thermo Fisher Scientific K.K.) with bovine serum albumin as the standard. SDS/PAGE analysis was carried out according to the Laemmli method 50. Internal terminal aa sequencing of the ~ 35‐kDa band was performed with nanoLC‐MS/MS on a Xevo QTOF MS system (Waters Corp., Milford, MA, USA) as described previously 25. The enzyme gene was identified on proteinlynx global server, version 2.3 (Waters) using the ORF database based on the NGS results.

### Nucleotide and peptide sequence accession numbers

The nucleotide sequences of the 16S rDNA of strain RD4668 and the LyPls‐PLD gene, designated *lpls‐pld*, were deposited in the DDBJ database under the accession numbers AB873024 and AB874601, respectively.

### Functional expression and purification of recombinant LyPls‐PLD

An expression vector pET24a(+) (Merck KGaA, Darmstadt, Germany) was used, and two recombinant plasmid vectors, pET24a/*lpls‐pld mat* (without His tag) and pET24a/*lpls‐pld mat*_his (with His tag), were constructed to produce active LyPls‐PLD as previously reported 28. The recombinant vector pET24a/*lpls‐pld mat* carried the gene for mature LyPls‐PLD, *lpls‐pld mat*, between the *Nde*I and *Eco*RI sites of pET24a(+), while pET24a/*lpls‐pld mat*_his contained *lpls‐pld mat* between the *Nde*I site and a C‐terminal His6 tag. To construct pET24a/*lpls‐pld mat*, PCR primers were designed based on the ORF of *lpls‐pld* identified in the ORF database. The forward primer 5′‐ttcatatg *acc*accagaacgacagacaatc‐3′ containing the N‐terminal codon (*Nde*I, single underline; Thr, italics) and the reverse primer 5′‐ttgaattctcatccgcacggatcgacgccc‐3′ containing the stop codon and *Eco*RI site were used. One Shot BL21 (DE3) Chemically Competent *E. coli* (Thermo Fisher Scientific K.K.) cells were transformed with the recombinant expression vectors, after which the transformants were selected on Luria–Bertani (LB) agar plates containing 50 μg·mL^−1^ ampicillin (LBA) and then confirmed by colony PCR. Each transformant was screened for LyPls‐PLD activity during 5 mL cultivation in LBA broth, and those exhibiting the highest activity were selected. To construct pET24a/*lpls‐pld mat*_his, His6 tag was joined to the C‐terminal aa sequence of LyPls‐PLD by inverse PCR as previously reported 48 using pET24a/*lpls‐pld mat* as a template and the inverse primers 5′‐caccaccaccaccaccactg‐3′ and 5′‐tccgcacggatcgacgcccgg‐3′. SHuffle^**®**^ T7 Express Competent *E. coli* cells (New England Biolabs Japan, Tokyo, Japan) were transformed with the recombinant expression vectors, after which the transformants were selected on LB agar plates containing 30 μg·mL^−1^ kanamycin (LBK). Finally, a transformant exhibiting the highest activity was selected as above. Shuffle T7 *E. coli* cells harboring pET24a/*lpls‐pld mat*_his were inoculated into a test tube containing 5 mL LBK seed medium and cultivated overnight at 30 °C with shaking (160 strokes·min^−1^). Thereafter, 1% (v/v) inocula were transferred to 500‐mL flasks containing 100 mL of LBK fermentation medium and cultivated at 30°C with shaking (160 rpm). After 24 h, 0.4 mm isopropyl‐β‐d‐thiogalactopyranoside was added, and the cultures were continued for an additional 4 h at 30°C. Two hundred milliliters of the recombinant *E. coli* cell cultures were then centrifuged (18 800 ***g***, 20 min), and the resulting cell paste (~ 2 g wet weight) was washed twice and suspended in buffer A. The cells were disrupted using a sonicator UD‐201 (Tomy Seiko Co., Ltd., Tokyo, Japan; 100 W, 20 kHz, 10 min, on ice) and centrifuged at 21 800 ***g*** for 20 min. The cell‐free extracts (cfe) were then loaded onto a HisTrap HP column (5 mL) equilibrated with 0.5 m NaCl, 5 mm imidazole/50 mm buffer A. After washing, the proteins were eluted with a linear gradient (15 CV) of 5 to 500 mm imidazole in the same buffer at 1.5 cm·min^−1^. Fractions exhibiting high specific activity with high purity were pooled followed by buffer exchange into buffer A as described above and used for subsequent investigation.

### Enzymatic characterization

The purified rLyPls‐PLD was used for enzymatic characterization. Except for LyPlsCho, LyPlsEtn, SM, and GPC, the insoluble substrates such as PlsCho, PlsEtn, and PLs were dispersed in distilled water by vortexing and sonication. The liposomal substrate composed of 1 mM POPC/0.4 mM LyPlsCho was prepared according to the hydration method and used as liposomal substrate 26, 51. The enzyme activity toward each substrate was assayed under standard assay condition I. The effect of metal ions on enzyme activity was investigated under standard assay condition I with the same buffer containing 2 mm metal ion, EDTA, or 1 mm inhibitor. Inhibitors assessed were DTT, 2ME, IAA, and PMSF. The effect of Triton X‐100 concentration ([Triton X‐100]) and Ca^2+^ concentration ([Ca^2+^]) on enzyme activity was investigated under standard assay condition I. The effect of substrate analogs on LyPls‐PLD activity was examined under standard assay condition I containing 0.4 mm LyPlsCho and 4 mm substrate analog. All experiments were carried out three times independently.

Each buffer (sodium acetate, MES‐NaOH, BisTris/HCl, Tris/HCl, and glycine‐NaOH) was used to investigate the effect of pH on enzyme activity and stability. The optimum pH was examined under standard assay condition I (37 °C, 1 min) with 0.4 mm LyPlsCho and 2 mm CaCl_2_ in 80 mm of each buffer. To determine pH stability, the enzyme sample was incubated at 4 °C for 4 h in 50 mm of each buffer. The residual activity was assayed under standard assay condition I (50 °C, pH 8.0 for 1 min: standard assay condition II). The optimum temperature was determined by measuring enzyme activity at each temperature under standard assay condition II. To determine thermal stability, the enzyme sample was incubated at each temperature for 60 min in 20 mm Tris/HCl buffer (pH 8.0), and the residual activity was assayed under standard assay condition II. All experiments were independently carried out three times.

### Steady‐state kinetics

For rLyPls‐PLD, the initial velocity (*v*) of the enzymatic reaction for 30 s (LyPlsCho) or 3 min (LyPlsEtn) was determined at several substrate concentrations ([LyPlsCho]) under standard assay condition II. The concentration of rLyPls‐PLD in the reaction mixture was held constant at 0.508 μg·L^−1^ (14.8 nm) for LyPlsCho and 148 nm for LyPlsEtn calculated as a monomeric protein with molecular mass of 34 405.58 Da with a His6 tag and Met start codon, without the signal peptide. The corresponding *v* versus [LyPlsCho] plot was evaluated using the Michaelis–Menten equation. For the WT enzyme, the kinetic constants *K*_m_, *V*_max_, and *k*_cat_ were determined using nonlinear regression (kaleidagraph, Synergy Software, Reading, PA, USA). For the mutant enzyme, F211A, kinetic constants were determined by extrapolation using the Hanes–Woolf plot ([S]/*v* versus [S]) by linear regression (kaleidagraph). The *K*_m_ and *V*_max_ were determined from the *x*‐intercept and slope of the regression line, respectively.

### Homology modeling of LyPls‐PLD and docking model with substrate analog

Based on a template of five proteins (Protein Data Bank code, 3L12, 4R7O, 2OOG, 3QVQ, 3KS6), the homology model of LyPls‐PLD was created using an HHPRED search (http://toolkit.tuebingen.mpg.de/hhpred) 52 and Modeller 9.16 (http://toolkit.tuebingen.mpg.de/modeller) 53. VERIFY3D (http://services.mbi.ucla.edu/Verify_3D/) 54 was used to assess the quality of the predicted models, which were drawn using molfeat Version 5.1.0.24 (FiatLux Corp., Tokyo, Japan). The docking model between LyPls‐PLD and LyPlsCho analog with a short chain (C3)‐alkenyl ether bond at *sn*‐1 was simulated using AutoDock 55.

### Site‐directed mutagenesis

We speculated that the highly conserved residues of H46, E73, D75, H88, E175, F211, and W282 in LyPls‐PLD are likely concerned with catalytic function. Moreover, based on the substrate analog docking simulation analysis, we considered that E50, A55, and N56 located near the nitrogen atom of choline in the substrate analog may be involved in recognizing the substrate head group. We then tried to generate the mutant enzymes of rLyPls‐PLD, H46A/R, E50D, A55N/D/E, N56E/D/Q, E73A/R, D75A/R, H88A/R, E175A/R, F211A/R, and W282A/R using pET24a/*lpls‐pld mat*_his as the template and our previously described inverse PCR method 15. The PCR product was treated with *Dpn*I to digest the parental DNA template and select for mutation‐containing synthesized DNA. The pET24 vector DNA incorporating the desired mutations was then transformed into Shuffle T7 *E. coli* cells. The mutants were selected and cultured in 5 mL LBK broth as described for rLyPls‐PLD production. The variant enzymes were purified using the HisTrap column as above.

## Author contributions

Each author contributed to the article as follows: DS conceived the study and drafted the manuscript. DS, SS, and HM participated in its design and coordination. YM carried out purification, characterization, and gene cloning. YM, NK, TO, and KM performed mutagenesis analysis, expression, and structure modeling studies. YM, NK, TO, SS, KM, and DS analyzed data. DS wrote this paper. All authors read and approved the final manuscript.

## Supporting information


**Fig. S1.** SDS/PAGE analysis of purified enzyme (A) and rLyPls‐PLD produced using transformed *E. coli* (B).

**Fig. S2**. Michaelis–Menten plot of steady‐state kinetics.

**Fig. S3**. Predicted catalytic mechanism of LyPls‐PLD.

**Table S1.** Contigs assembled by Velvet and ORF prediction by getorf.

## References

[1] Wu L‐C , Pfeiffer DR , Calhoon EA , Madiai F , Marcucci G , Liu S and Jurkowitz MS (2011) Purification, identification, and cloning of lysoplasmalogenase, the enzyme that catalyzes hydrolysis of the vinyl ether bond of lysoplasmalogen. J Biol Chem 286, 24916–24930. 21515882 10.1074/jbc.M111.247163 PMC3137066

[2] Nagan N and Zoeller RA (2001) Plasmalogens: biosynthesis and functions. Prog Lipid Res 40, 199–229. 11275267 10.1016/s0163-7827(01)00003-0

[3] Jurkowitz MS , Patel A , Wu L‐C , Krautwater A , Pfeiffer DR and Bell CE (2015) The YhhN protein of *Legionella pneumophila* is a lysoplasmalogenase. Biochim Biophys Acta 1848, 742–751. 25445671 10.1016/j.bbamem.2014.11.011 PMC4282143

[4] Nakamura K , Igarashi K , Ide K , Ohkawa R , Okubo S , Yokota H , Masuda A , Oshima N , Takeuchi T and Nangaku M (2008) Validation of an autotaxin enzyme immunoassay in human serum samples and its application to hypoalbuminemia differentiation. Clin Chim Acta 388, 51–58. 17963703 10.1016/j.cca.2007.10.005

[5] Wood PL , Mankidy R , Ritchie S , Heath D , Wood JA , Flax J and Goodenowe DB (2010) Circulating plasmalogen levels and Alzheimer disease assessment scale‐cognitive scores in Alzheimer patients. J Psychiatry Neurosci 35, 59–62. 20040248 10.1503/jpn.090059 PMC2799506

[6] Wang S , Zhang S , Liou L‐C , Ren Q , Zhang Z , Caldwell GA , Caldwell KA and Witt SN (2014) Phosphatidylethanolamine deficiency disrupts α‐synuclein homeostasis in yeast and worm models of Parkinson disease. Proc Natl Acad Sci USA 111, E3976–E3985. 25201965 10.1073/pnas.1411694111 PMC4183298

[7] Nishimukai M , Maeba R , Ikuta A , Asakawa N , Kamiya K , Yamada S , Yokota T , Sakakibara M , Tsutsui H , Sakurai T *et al* (2014) Serum choline plasmalogens—those with oleic acid in *sn*−2—are biomarkers for coronary artery disease. Clin Chim Acta 437, 147–154. 25068205 10.1016/j.cca.2014.07.024

[8] Sakasegawa S‐I , Maeba R , Murayama K , Matsumoto H and Sugimori D (2015) Hydrolysis of plasmalogen by phospholipase A_1_ from *Streptomyces albidoflavus* for early detection of dementia and arteriosclerosis. Biotechnol Lett 38, 109–116. 26354853 10.1007/s10529-015-1955-5

[9] Jurkowitz MS , Horrocks LA and Litsky ML (1999) Identification and characterization of alkenyl hydrolase (lysoplasmalogenase) in microsomes and identification of a plasmalogen‐active phospholipase A_2_ in cytosol of small intestinal epithelium. Biochim Biophys Acta 1437, 142–156. 10064898 10.1016/s1388-1981(99)00013-x

[10] Kramer RM and Deykin D (1983) Arachidonoyl transacylase in human platelets. Coenzyme A‐independent transfer of arachidonate from phosphatidylcholine to lysoplasmenylethanolamine. J Biol Chem 258, 13806–13811. 6417134

[11] Wykle RL and Schremmer JM (1974) A lysophospholipase D pathway in the metabolism of ether‐linked lipids in brain microsomes. J Biol Chem 249, 1742–1746. 4855486

[12] Warner HR and Lands WE (1961) The metabolism of plasmalogen: enzymatic hydrolysis of the vinyl ether. J Biol Chem 236, 2404. 13783189

[13] Gunawan J , Vierbuchen M and Debuch H (1979) Studies on the hydrolysis of 1‐alk‐1′‐enyl‐*sn*‐glycero‐3‐phosphoethanolamine by microsomes from myelinating rat brain. Hoppe Seylers Z Physiol Chem 360, 971–978. 488919 10.1515/bchm2.1979.360.2.971

[14] Wolf R and Gross R (1985) Semi‐synthetic approach for the preparation of homogeneous plasmenylethanolamine utilizing phospholipase D from *Streptomyces chromofuscus* . J Lipid Res 26, 629–633. 4020302

[15] Matsumoto Y and Sugimori D (2015) Substrate recognition mechanism of *Streptomyces* phospholipase D and enzymatic measurement of plasmalogen. J Biosci Bioeng 120, 372–379. 25900053 10.1016/j.jbiosc.2015.02.020

[16] Mitaku S , Hirokawa T and Tsuji T (2002) Amphiphilicity index of polar amino acids as an aid in the characterization of amino acid preference at membrane‐water interfaces. Bioinformatics 18, 608–616. 12016058 10.1093/bioinformatics/18.4.608

[17] Munk C , Lapidus A , Copeland A , Jando M , Mayilraj S , Glavina Del Rio T , Nolan M , Chen F , Lucas S , Tice H *et al* (2009) Complete genome sequence of *Stackebrandtia nassauensis* type strain (LLR‐40K‐21). Stand Genomic Sci 1, 234–241. 10.4056/sigs.47643 PMC3035245 21304662

[18] Shi L , Liu J‐F , An X‐M and Liang D‐C (2008) Crystal structure of glycerophosphodiester phosphodiesterase (GDPD) from *Thermoanaerobacter tengcongensis*, a metal ion‐dependent enzyme: insight into the catalytic mechanism. Proteins: Struct, Funct, Bioinf 72, 280–288. 10.1002/prot.21921 18214974

[19] Uesugi Y and Hatanaka T (2009) Phospholipase D mechanism using *Streptomyces* PLD. Biochim Biophys Acta 1791, 962–969. 19416643 10.1016/j.bbalip.2009.01.020

[20] Punta M , Coggill PC , Eberhardt RY , Mistry J , Tate J , Boursnell C , Pang N , Forslund K , Ceric G , Clements J *et al* (2012) The Pfam protein families database. Nucleic Acids Res 40, D290–D301. 22127870 10.1093/nar/gkr1065 PMC3245129

[21] Ogino C , Kanemasu M , Hayashi Y , Kondo A , Shimizu N , Tokuyama S , Tahara Y , Kuroda S , Tanizawa K and Fukuda H (2004) Over‐expression system for secretory phospholipase D by *Streptomyces lividans* . Appl Microbiol Biotechnol 64, 823–828. 14740197 10.1007/s00253-003-1552-8

[22] Lammertyn E and Anné J (1998) Modifications of *Streptomyces* signal peptides and their effects on protein production and secretion. FEMS Microbiol Lett 160, 1–10. 9495006 10.1111/j.1574-6968.1998.tb12882.x

[23] Iwasaki Y , Niwa S , Nakano H , Nagasawa T and Yamane T (1994) Purification and properties of phosphatidylinositol‐specific phospholipase C from *Streptomyces antibioticus* . Biochim Biophys Acta 1214, 221–228. 7918603

[24] Ogino C , Negi Y , Matsumiya T , Nakaoka K , Kondo A , Kuroda SI , Tokuyama S , Kikkawa U , Yamane T and Fukuda H (1999) Purification, characterization, and sequence determination of phospholipase D secreted by *Streptoverticillium cinnamoneum* . J Biochem (Tokyo) 125, 263–269. 9990122 10.1093/oxfordjournals.jbchem.a022282

[25] Sugimori D , Kano K and Matsumoto Y (2012) Purification, characterization, molecular cloning and extracellular production of a phospholipase A_1_ from *Streptomyces albidoflavus* NA297. FEBS Open Bio 2, 318–327. 10.1016/j.fob.2012.09.006 PMC3678127 23772365

[26] Matsumoto Y , Mineta S , Murayama K and Sugimori D (2013) A novel phospholipase B from *Streptomyces* sp. NA684: purification, characterization, gene cloning, extracellular production, and prediction of the catalytic residues. FEBS J 280, 3780–3796. 23731334 10.1111/febs.12366

[27] Sugimori D , Ogasawara J , Okuda K and Murayama K (2014) Purification, characterization, molecular cloning, and extracellular production of a novel bacterial glycerophosphocholine cholinephosphodiesterase from *Streptomyces sanglieri* . J Biosci Bioeng 117, 422–430. 24211038 10.1016/j.jbiosc.2013.10.004

[28] Mineta S , Murayama K and Sugimori D (2015) Characterization of glycerophosphoethanolamine ethanolaminephosphodiesterase from *Streptomyces sanglieri* . J Biosci Bioeng 119, 123–130. 25135787 10.1016/j.jbiosc.2014.07.005

[29] Larson TJ , Ehrmann M and Boos W (1983) Periplasmic glycerophosphodiester phosphodiesterase of *Escherichia coli*, a new enzyme of the *glp* regulon. J Biol Chem 258, 5428–5432. 6304089

[30] Imamura S and Horiuti Y (1979) Purification of *Streptomyces chromofuscus* phospholipase D by hydrophobic affinity chromatography on palmitoyl cellulose. J Biochem 85, 79–95. 762053 10.1093/oxfordjournals.jbchem.a132334

[31] Fu J , Huang H , Meng K , Yuan T , Yao B , Shi Y and Ouyang P (2008) A novel cold‐adapted phospholipase A_1_ from *Serratia* sp. xjF1: gene cloning, expression and characterization. Enzyme Microb Technol 42, 187–194. 22578870 10.1016/j.enzmictec.2007.09.004

[32] Mander P , Simkhada JR , Cho SS , Park SJ , Choi HS , Lee HC , Sohng JK and Yoo JC (2009) A novel Ca ^2+^‐dependent phospholipase D from *Streptomyces tendae*, possessing only hydrolytic activity. Arch Pharm Res 32, 1461–1467. 19898811 10.1007/s12272-009-2017-0

[33] Nakazawa Y , Sagane Y , Kikuchi T , Uchino M , Nagai T , Sato H , Toeda K and Takano K (2010) Purification, biochemical characterization, and cloning of phospholipase D from *Streptomyces racemochromogenes* strain 10‐3. Protein J 29, 598–608. 21082226 10.1007/s10930-010-9292-y

[34] Qin C and Wang X (2002) The *Arabidopsis* phospholipase D family. Characterization of a calcium‐independent and phosphatidylcholine‐selective PLDζ1 with distinct regulatory domains. Plant Physiol 128, 1057–1068. 11891260 10.1104/pp.010928 PMC152217

[35] Wan SB , Wang W , Wen PF , Chen JY , Kong WF , Pan QH , Zhan JC , Tian L , Liu HT and Huang WD (2007) Cloning of phospholipase D from grape berry and its expression under heat acclimation. J Biochem Mol Biol 40, 595–603. 17669277 10.5483/bmbrep.2007.40.4.595

[36] Simkhada JR , Lee HJ , Jang SY , Cho SS , Park EJ , Sohng JK and Yoo JC (2009) A novel alkalo‐ and thermostable phospholipase D from *Streptomyces olivochromogenes* . Biotechnol Lett 31, 429–435. 19039525 10.1007/s10529-008-9890-3

[37] Yang H and Roberts MF (2002) Cloning, overexpression, and characterization of a bacterial Ca^2+^‐dependent phospholipase D. Protein Sci 11, 2958–2968. 12441393 10.1110/ps.0225302 PMC2373738

[38] Yang H and Roberts MF (2003) Phosphohydrolase and transphosphatidylation reactions of two *Streptomyces* phospholipase D enzymes: covalent versus noncovalent catalysis. Protein Sci 12, 2087–2098. 12931007 10.1110/ps.03192503 PMC2324005

[39] Felts RL , Reilly TJ and Tanner JJ (2006) Structure of *Francisella tularensis* AcpA: prototype of a unique superfamily of acid phosphatases and phospholipases C. J Biol Chem 281, 30289–30298. 16899453 10.1074/jbc.M606391200

[40] Kokusho Y , Kato S , Machida H and Iwasaki S (1987) Purification and properties of phospholipase D from *Actinomadura* sp. strain No. 362. Agric Biol Chem 51, 2515–2524.

[41] Sugimori D , Matsumoto Y , Tomita Y , Murayama K and Ogino C (2012) Kinetic characterization and Mg^2+^ enhancement of *Streptomyces griseocarneus* sphingomyelinase C produced by recombinant *Streptomyces lividans* . Protein Expr Purif 81, 151–156. 22027400 10.1016/j.pep.2011.10.004

[42] Okawa Y and Yamaguchi T (1975) Studies on phospholipases from *Streptomyces* . J Biochem (Tokyo) 78, 537–545. 5411 10.1093/oxfordjournals.jbchem.a130938

[43] Simkhada JR , Cho SS , Lee HJs and Yoo JC (2007) Purification and biochemical properties of phospholipase D (PLD57) produced by *Streptomyces* sp. CS‐57. Arch Pharm Res 30, 1302–1308. 18038909 10.1007/BF02980271

[44] Sugimori D (2009) Purification, characterization, and gene cloning of sphingomyelinase C from *Streptomyces griseocarneus* NBRC13471. J Biosci Bioeng 108, 293–298. 19716517 10.1016/j.jbiosc.2009.04.013

[45] Suzuki A , Kakuno K , Iwasaki Y , Yamane T and Yamane T (1999) Crystallization and preliminary X‐ray diffraction studies of phospholipase D from *Streptomyces antibioticus* . Acta Crystallogr Sect D 55, 317–319. 10089437 10.1107/S0907444998010592

[46] Qin H‐M , Yamamura A , Miyakawa T , Kataoka M , Nagai T , Kitamura N , Urano N , Maruoka S , Ohtsuka J and Nagata K (2014) Structure of conjugated polyketone reductase from *Candida parapsilosis* IFO 0708 reveals conformational changes for substrate recognition upon NADPH binding. Appl Microbiol Biotechnol 98, 243–249. 23828603 10.1007/s00253-013-5073-9

[47] Iwasaki Y , Horiike S , Matsushima K and Yamane T (1999) Location of the catalytic nucleophile of phospholipase D of *Streptomyces antibioticus* in the C‐terminal half domain. Eur J Biochem 264, 577–581. 10491106 10.1046/j.1432-1327.1999.00669.x

[48] Hirano Y , Chonan K , Murayama K , Sakasegawa S‐I , Matsumoto H and Sugimori D (2016) *Syncephalastrum racemosum* amine oxidase with high catalytic efficiency toward ethanolamine and its application in ethanolamine determination. Appl Microbiol Biotechnol 100, 3999–4013. 26691518 10.1007/s00253-015-7198-5

[49] Pospiech A and Neumann B (1995) A versatile quick‐prep of genomic DNA from gram‐positive bacteria. Trends Genet 11, 217–218. 7638902 10.1016/s0168-9525(00)89052-6

[50] Laemmli UK (1970) Cleavage of structural proteins during the assembly of the head of bacteriophage T4. Nature 227, 680–685. 5432063 10.1038/227680a0

[51] Tanaka H and Kobayashi T (2011) Characterization of a 62‐kilodalton acidic phospholipid‐binding protein isolated from the edible mushroom *Pleurotus ostreatus* . J Health Sci 57, 99–106.

[52] Söding J (2005) Protein homology detection by HMM‐HMM comparison. Bioinformatics 21, 951–960. 15531603 10.1093/bioinformatics/bti125

[53] Sali A , Potterton L , Yuan F , van Vlijmen H and Karplus M (1995) Evaluation of comparative protein modeling by MODELLER. Proteins: Struct, Funct, Bioinf 23, 318–326. 10.1002/prot.340230306 8710825

[54] Liithy R , Bowie JU and Eisenberg D (1992) Assessment of protein models with three‐dimensional profiles. Nature 356, 83–85. 1538787 10.1038/356083a0

[55] Trott O and Olson AJ (2010) AutoDock Vina: improving the speed and accuracy of docking with a new scoring function, efficient optimization, and multithreading. J Comput Biol 31, 455–461. 10.1002/jcc.21334 PMC3041641 19499576

[56] Sakagami H , Aoki J , Natori Y , Nishikawa K , Kakehi Y , Natori Y and Arai H (2005) Biochemical and molecular characterization of a novel choline‐specific glycerophosphodiester phosphodiesterase belonging to the nucleotide pyrophosphatase/phosphodiesterase family. J Biol Chem 280, 23084–23093. 15788404 10.1074/jbc.M413438200

