# Supplementary material for: Molecular cloning, heterologous expression, and enzymatic characterization of lysoplasmalogen‐specific phospholipase D from Thermocrispum sp
Source: FEBS Open Bio. 2016 Oct 17;6(11):1113–30. doi: 10.1002/2211-5463.12131 (PMC5095149; doi:10.1002/2211-5463.12131)
Supplement: Supplementary file 1 — Fig. S1. SDS/PAGE analysis of purified enzyme (A) and rLyPls‐PLD produced using transformed E. coli (B). [file FEB4-6-1113-s001.pptx]

## Slide 1
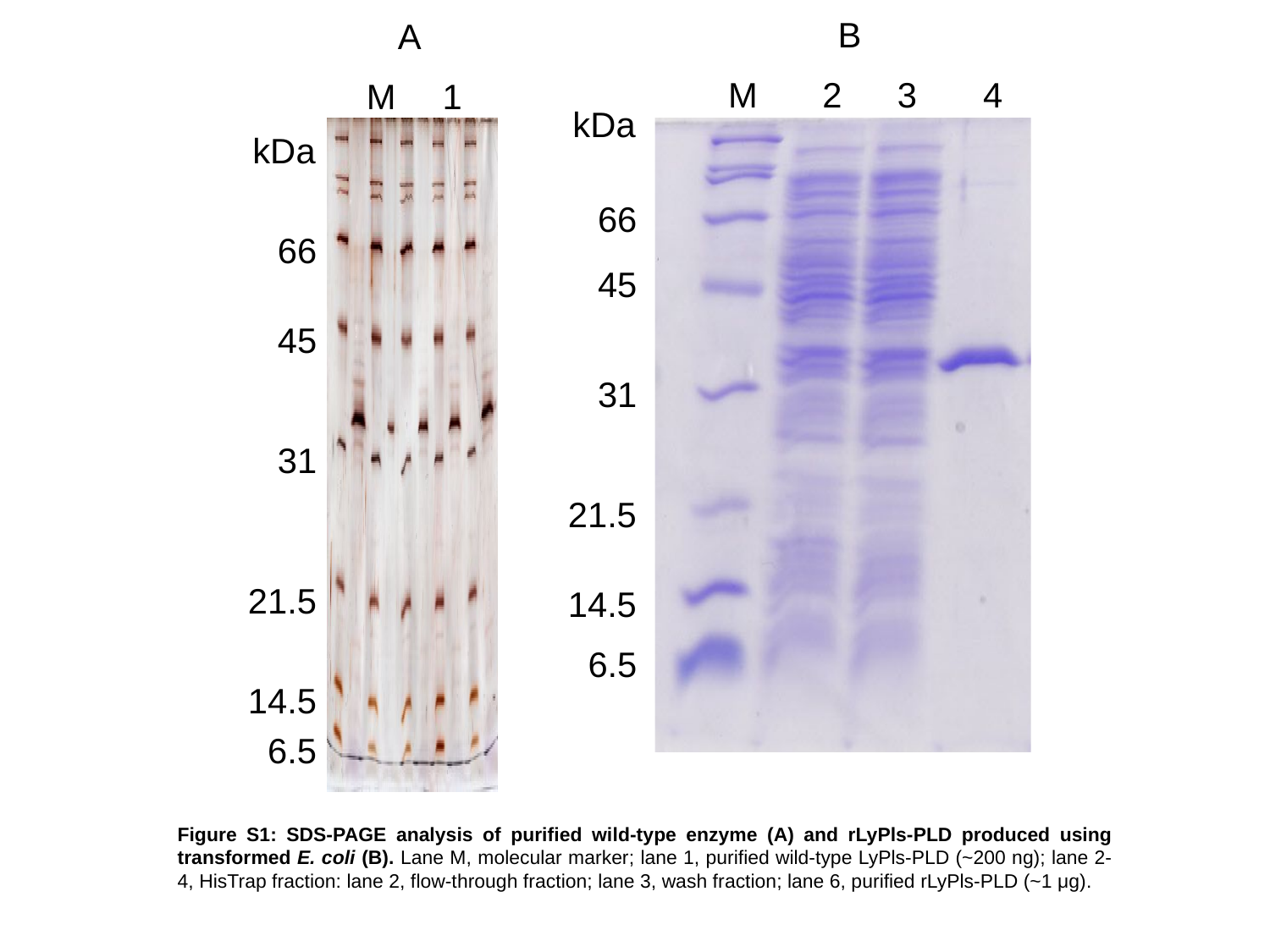

B
A
M
2
3
4
M
1
kDa
kDa
66
66
45
45
31
31
21.5
21.5
14.5
6.5
14.5
6.5
Figure S1: SDS-PAGE analysis of purified wild-type enzyme (A) and rLyPls-PLD produced using transformed E. coli (B). Lane M, molecular marker; lane 1, purified wild-type LyPls-PLD (~200 ng); lane 2-4, HisTrap fraction: lane 2, flow-through fraction; lane 3, wash fraction; lane 6, purified rLyPls-PLD (~1 μg).
