# Supplementary material for: Molecular cloning, heterologous expression, and enzymatic characterization of lysoplasmalogen‐specific phospholipase D from Thermocrispum sp
Source: FEBS Open Bio. 2016 Oct 17;6(11):1113–30. doi: 10.1002/2211-5463.12131 (PMC5095149; doi:10.1002/2211-5463.12131)
Supplement: Supplementary file 2 — Fig. S2. Michaelis–Menten plot of steady‐state kinetics. [file FEB4-6-1113-s002.pptx]

## Slide 1
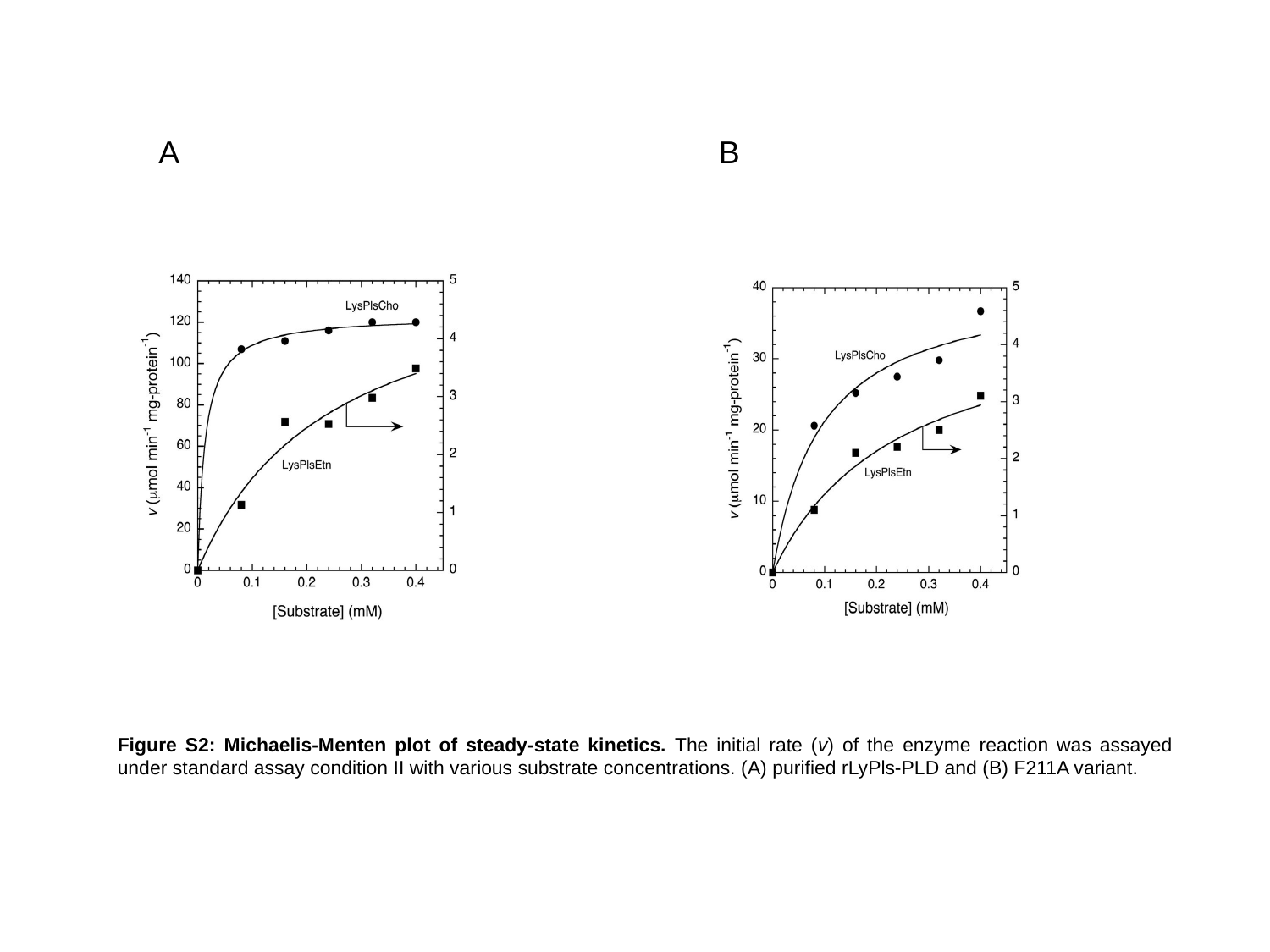

A
B
Figure S2: Michaelis-Menten plot of steady-state kinetics. The initial rate (v) of the enzyme reaction was assayed under standard assay condition II with various substrate concentrations. (A) purified rLyPls-PLD and (B) F211A variant.
