# Supplementary figures and images for: Molecular cloning, heterologous expression, and enzymatic characterization of lysoplasmalogen‐specific phospholipase D from Thermocrispum sp
Source: FEBS Open Bio. 2016 Oct 17;6(11):1113–30. doi: 10.1002/2211-5463.12131 (PMC5095149; doi:10.1002/2211-5463.12131)

## Slide 1
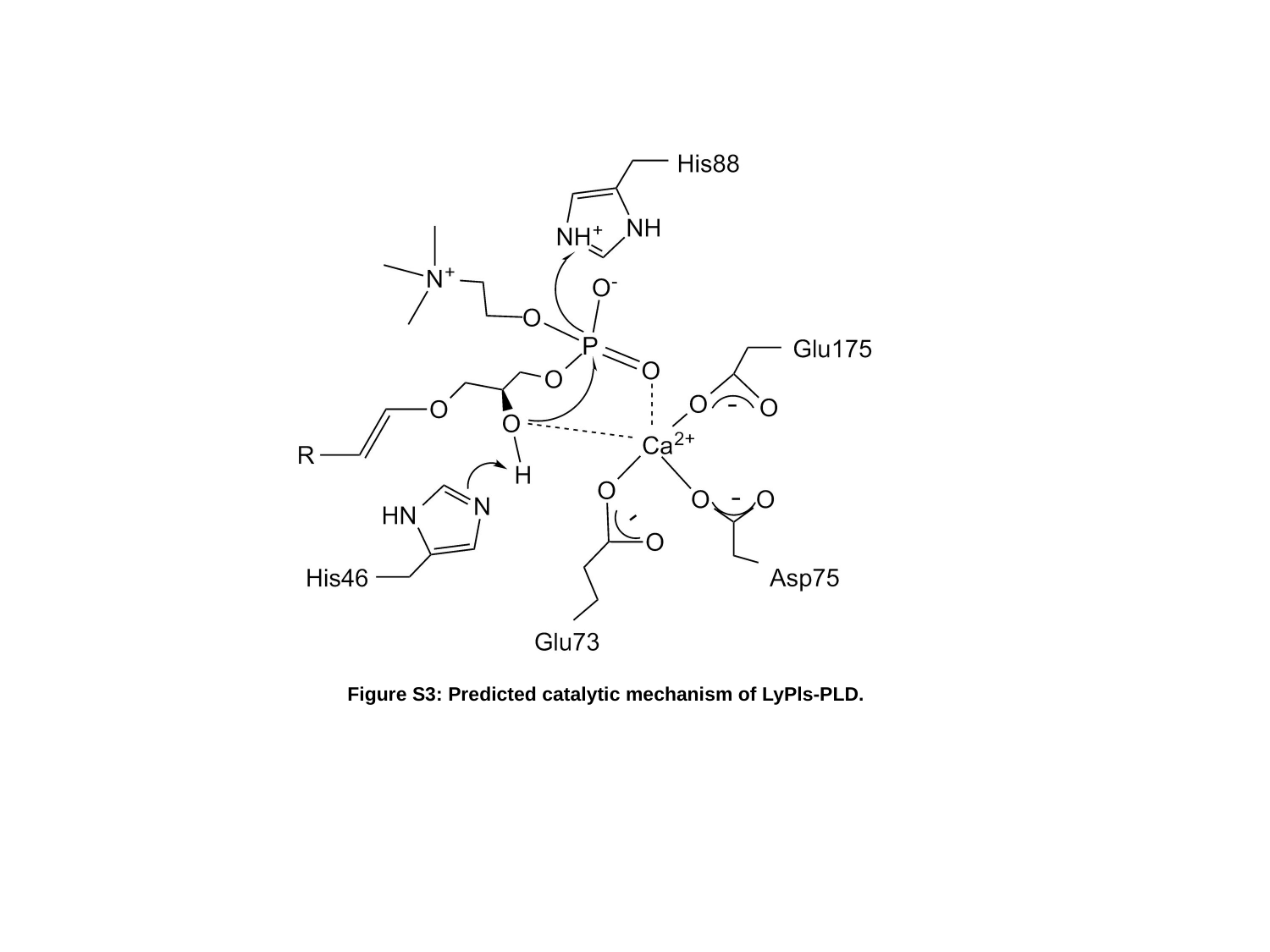

Figure S3: Predicted catalytic mechanism of LyPls-PLD.

Supplement: Supplementary file 3 — Fig. S3. Predicted catalytic mechanism of LyPls‐PLD. [file FEB4-6-1113-s003.pptx]
