# Supplementary material for: Molecular cloning, heterologous expression, and enzymatic characterization of lysoplasmalogen‐specific phospholipase D from Thermocrispum sp
Source: FEBS Open Bio. 2016 Oct 17;6(11):1113–30. doi: 10.1002/2211-5463.12131 (PMC5095149; doi:10.1002/2211-5463.12131)
Supplement: Supplementary file 4 — Table S1. Contigs assembled by Velvet and ORF prediction by getorf. [file FEB4-6-1113-s004.docx]

**Table S1. Contigs assembled by Velvet and ORF prediction by getorf**

| Hash length | 75 |
| --- | --- |
| Using reads | 60,903,577/68,428,922 |
| Number of contigs | 32 |
| Total length (bp) | 4,582,032 |
| Average length (bp) | 143,189 |
| Maximum contig length (bp) | 1,055,262 |
| Predicted ORFs | 67,013 |

ORF = open reading frame
